# Projected shifts in climatic suitability of olive (*Olea europaea* L.) in the Mediterranean

**DOI:** 10.3389/fpls.2026.1743577

**Published:** 2026-03-13

**Authors:** Giacomo Belvisi, Giorgia Tranchina

**Affiliations:** Department of Agricultural, Food and Forest Sciences, University of Palermo, Palermo, Italy

**Keywords:** aridity index, crop suitability, future climate scenario, MaxEnt, soil type, species distribution models

## Abstract

**Context:**

Climate change is reconfiguring the spatial structure of agricultural landscapes worldwide. The Mediterranean Basin, a hotspot of change and the core region for olive cultivation, faces significant shifts in the distribution of this economically and culturally pivotal crop, with profound implications for landscape ecology, ecosystem services, and sustainability.

**Objective:**

This study aims to assess current and future climatic suitability for olive cultivation (*Olea europaea* L.) across the Mediterranean Basin, identifying potential shifts in suitable areas under contrasting climate scenarios while explicitly accounting for model complexity, transferability, and extrapolation uncertainty.

**Methods:**

We applied a species distribution modeling framework based on MaxEnt using 7,490 presence records and a set of bioclimatic predictors at 30 arc-second resolution. A baseline MaxEnt model was first developed to ensure comparability with previous basin-scale assessments. Model calibration and transferability were subsequently refined using the kuenm package, exploring multiple combinations of feature classes, regularization multipliers, and variable subsets. Future projections were generated under CMIP6 SSP1-2.6 and SSP5-8.5 scenarios for mid- and late-century periods. Extrapolation risk was evaluated using Mobility-Oriented Parity (MOP) analysis, while soil and aridity constraints were explored through *post-hoc* analyses.

**Results:**

Minimum temperature of the coldest month (Bio6) emerged as the dominant predictor of olive suitability. Model projections consistently indicate a northward expansion of climatically suitable areas, particularly in France, driven by the relaxation of winter temperature constraints. Traditional olive-growing regions in southern Europe and North Africa remain broadly suitable but exhibit increasing exposure to climatic stress related to heat, reduced chilling, and water scarcity. *Post-hoc* analyses suggest that soil type and aridity indices alone are insufficient to delineate suitability patterns at this spatial resolution.

**Conclusions:**

Our results reveal a substantial reorganization of Mediterranean olive-growing landscapes under future climate change. While new opportunities may emerge in currently marginal regions, climatic suitability does not automatically translate into agronomic viability. Adaptation strategies in traditional areas and cautious planning in emerging regions will be essential. By integrating basin-scale modeling with rigorous calibration and extrapolation diagnostics, this study provides a robust and transparent framework to support adaptive planning and climate-resilient management of olive-based agroecosystems.

## 1 Introduction

The olive tree (*Olea europaea* L.) is one of the most emblematic components of Mediterranean landscapes, shaping their ecological structure, cultural identity, and agricultural economy for millennia (Carrión et al., 2010). Although the specific area of domestication remains debated, olive cultivation is presumed to have originated in the Middle East, within a region spanning Iran, Mesopotamia, and Palestine (Lavee, 1992), before gradually spreading throughout southern Europe and northern Africa, with their introduction to other regions of the world occurring only in more recent times (Besnard et al., 2018). Today, more than 95% of the global olive-growing areas, over 10 MLN ha, is still concentrated in Mediterranean Basin countries (FAOSTAT, 2025; **Supplementary Figure 1** in **Supplementary Information SI**), where olive orchards represent a dominant and multifunctional land-use system providing ecosystem services such as soil protection, carbon sequestration, and cultural heritage preservation (Ferrara and Ingemark, 2023).

The current geographical distribution of olive cultivation closely corresponds to regions characterized by Mediterranean-type climates (Csa and Csb in the Köppen-Geiger classification), defined by hot, dry summers and mild, wet winters (Kottek et al., 2006; Peel et al., 2007). These climatic conditions have historically shaped olive domestication and diffusion and remain central to its productivity and persistence (Zohary et al., 2012; Besnard et al., 2018). Olive cultivation exhibits exceptionally high genetic and varietal diversity, with over 1,200 formally named cultivars, several thousand minor locally adapted varieties, and wild genotypes. This diversity reflects millennia of domestication and farmer-driven selection across the Mediterranean Basin and represents a crucial reservoir of adaptive potential under climate change (Fanelli et al., 2022; Valeri et al., 2022; Mariotti et al., 2023). However, recent trends toward intensive and super-intensive systems have led to the dominance of a few highly productive cultivars. This homogenization threatens traditional varieties, which may be critical for future adaptation to climate change, pests, diseases, and breeding needs. The Mediterranean region is considered a climate-change hotspot, where rising temperatures, declining precipitation and increasing inter-annual variability are already evident and are projected to intensify over the coming decades (Giorgi, 2006; Giorgi and Lionello, 2008; IPCC, 2023). These changes are expected to affect key processes relevant to olive cultivation, including evapotranspiration demands, irrigation requirements, winter chilling accumulation, flowering phenology, pollination success, fruit set, and exposure to biotic stressors (Osborne et al., 2000; Quiroga and Iglesias, 2009; Salama et al., 2021). Despite the recognized vulnerability of Mediterranean agroecosystems, spatially explicit assessments of how climate change may alter the climatic suitability of olive cultivation across the entire Mediterranean Basin remain limited. Previous studies have investigated climate-related impacts on olive systems using a variety of approaches, including physiologically based demographic models (Moriondo et al., 2008; Ponti et al., 2014), agro-ecological zoning frameworks (Tanasijevic et al., 2014), and more recent species distribution modeling (SDM) applications at sub-national and continental scale (Ashraf et al., 2021; Yildiz et al., 2024). However, Mediterranean Basin assessments integrating up-to-date climate projections at 30-arc-second resolution, explicitly addressing the transferability of correlative models to future climatic conditions are still lacking.

Species distribution models based on the maximum entropy algorithm (Philipps et al., 2006) have become a standard tool for estimating climatic suitability using presence-only data and have been widely applied across ecological, environmental, and agricultural contexts. MaxEnt is a statistical inference procedure from incomplete information, initially originated in the area of statistical mechanics (Jaynes, 1957, 1982), applicated in the field of ecology only in the last two decades. It finds wide applications in species distribution modeling, ranging from herbaceous (Kumar et al., 2022), shrubby (Liu et al., 2018) or tree (Arenas-Castro et al., 2020; Ashraf et al., 2017; Hebbar et al., 2022; Özdel et al., 2024; Tóth and Végvári, 2016), to bacteria (Bosso et al., 2016a, 2016), mites (Li et al., 2024) and also pathogens together with its insect vector (Narouei-Khandan et al., 2016). Although it is not possible to guarantee accurate predictions, MaxEnt generates the least biased probability distribution predictions while adhering to known constraints on those distributions, minimizing the source of bias (Harte and Newman, 2014). While MaxEnt is well suited for exploratory and large-scale analysis, its application to future climate projections require careful calibration and explicit consideration of extrapolation risks associated with novel climatic conditions.

In this study, we combined a baseline MaxEnt modeling approach with a calibrated and transferability-aware framework implemented using the *kuenm* R package (Cobos et al., 2019). The baseline model was used to explore broad-scale climatic suitability patterns for olive cultivation across the Mediterranean Basin, while the *kuenm*-based workflow was subsequently applied to refine model calibration, explore model complexity, and explicitly assess extrapolation risks when projecting suitability under future climate scenarios.

The objectives of this study were to: (i) quantify the relative influence of climatic and topographic variables shaping the current distribution of olive cultivation across the Mediterranean Basin; and (ii) project future spatial patterns of climatic suitability under contrasting emission scenarios, identifying areas of potential expansion, contraction, and stability while accounting for model transferability and uncertainty. The novelty of this study lies primarily in its methodological framework, which integrates basin-wide extent, high-resolution climatic data, rigorous MaxEnt calibration, and explicit extrapolation diagnostics, providing a spatially consistent and transferable assessment of olive climatic suitability under future climate scenarios.

## 2 Material and methods

### 2.1 Analytical framework

**Figure 1:**
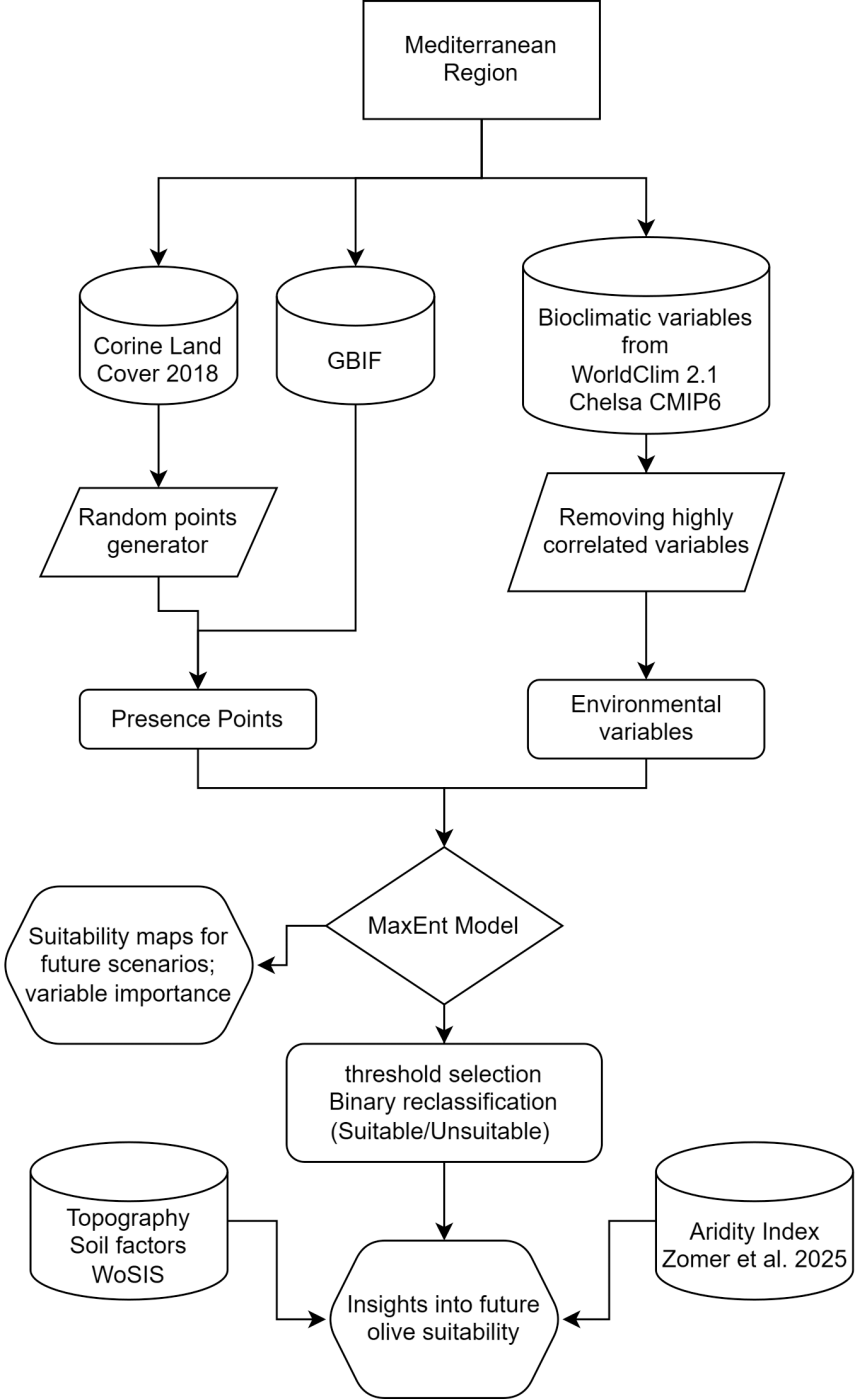
Flowchart illustrating the methodological framework adopted in this study for modeling current and future climatic suitability of olive in the Mediterranean Basin using MaxEnt, including data sources.

The workflow adopted in this study integrates spatially explicit species distribution modeling, climate change projections, and *post-hoc* landscape analyses to assess current and future climatic suitability for olive cultivation across the Mediterranean Basin (**Figure 1**). The framework combines a baseline MaxEnt modeling approach with a calibrated and transferability-oriented workflow implemented using the *kuenm* R package (Cobos et al., 2019). This dual strategy allows both comparability with previous studies and a more rigorous assessment of model complexity, uncertainty, and extrapolation risk under future climatic conditions.

Current climatic suitability was modelled using presence-only data and environmental predictors, while future projections were generated using CMIP6 climate scenarios. Model outputs were subsequently analyzed in relation to aridity and soil constraints to support ecological interpretation and management implications.

### 2.2 Study area

The study area encompasses the entire Mediterranean Basin, extending approximately from 27.20°N to 63.52°N latitude and from -18.17°E to 42.06°E longitude. A spatial mask covering approximately 18.58 million km^2^ was applied to delineate the modeling domain, clip environmental layers, and constrain species occurrence data. All spatial preprocessing was conducted in QGIS to ensure consistency across datasets.

### 2.3 Occurrence data

Presence data for *Olea europaea* were assembled using a dual-source approach to ensure comprehensive spatial coverage across the Mediterranean region. Following the methodology of Tóth and Végvári (2016), who employed the MaxEnt model to predict future winegrape distribution in Europe, we first accessed the Corine Land Cover, 2018 (CLC) dataset from the Copernicus platform to extract, with a spatial resolution of 25 ha (100 m), the olive grove areas (class 2.2.3). Within these areas 6,000 random presence points were generated using the QGIS random point generator. This initial point set represents presence data for Mediterranean countries participating in the Corine project: Portugal, Spain, France, Italy, Slovenia, Croatia, Albania, Greece, Türkiye, and Cyprus.

**Figure 2:**
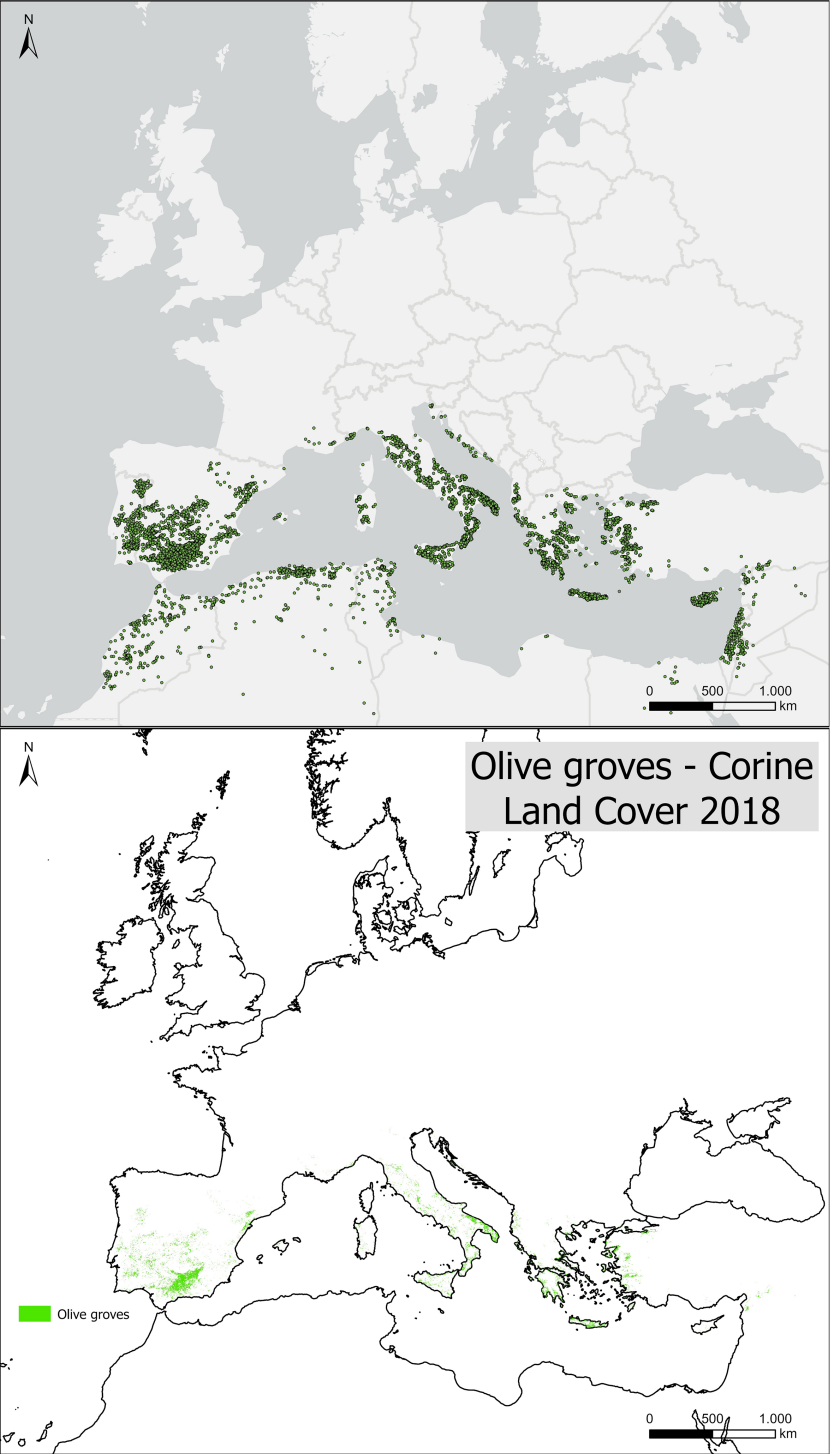
Occurrence points used in this study (top) and olive groves areas according to Corine Land Cover, 2018.

For Mediterranean countries not covered by CLC, occurrence records were retrieved from the Global Biodiversity Information Facility (GBIF, 2025) database (https://www.gbif.org/). Using the GBIF occurrences plugin in QGIS, records were obtained for Morocco, Algeria, Tunisia, Libya, Egypt, Israel, Palestine, Syria, Jordan, Lebanon, and Cyprus, yielding 1,490 presence points. All occurrence records were checked for spatial consistency and merged into a single dataset (**Figure 2**), available for download in the Supplementary Information as CSV files, formatted for direct use in the MaxEnt model.

### 2.4 Environmental predictors

Climatic suitability was modelled using bioclimatic and topographic predictors representing current (1970-2000), and future conditions (2041–2070 and 2071-2100). The nineteen bioclimatic variables and elevation were initially considered, consistent with recommendations for global and meso-scale analyses (Mackey and Lindenmayer, 2001). We examined two updated CMIP6 scenarios (IPCC, 2023), as detailed in **Table 1**.

**Table 1:** Description of climate change scenarios used in this study.

Scenario	Description
SSP1-2.6	Low GHG emissions (declining to net zero around 2070), followed by variable levels of net negative CO_2_ emissions
SSP5-8.5	Very high GHG emissions, with CO_2_ emissions roughly doubled from current levels by 2050

Current climate data (1970-2000) were obtained from WorldClim v.2.1 (Fick and Hijmans, 2017; https://www.worldclim.org/data/worldclim21.html) in GeoTiff format, at 30-arc-second resolution (approximately 0.93 x 0.93 km = 0.86 km² at the equator), together with elevation, derived from SRTM (Shuttle Radar Topography Mission). Future climate projections were based on a single high-resolution CMIP6 Earth System Model (MPI-ESM1-2-HR). This model was selected due to its documented ability to realistically reproduce key features of Mediterranean climate dynamics, including precipitation seasonality and large-scale atmospheric circulation patterns (Müller et al., 2018; Gutjahr et al., 2019). While multi-model ensembles are often recommended to quantify inter-model uncertainty, the use of a single, well-performing model is a common and accepted approach in regional-scale ecological niche modeling, particularly when the primary objective is to explore spatial patterns of climatic suitability and relative changes across scenarios rather than to provide absolute forecasts. Moreover, model uncertainty related to extrapolation under future climates was explicitly assessed through Mobility-Oriented Parity (MOP) analyses, allowing identification of areas where projections should be interpreted with caution. Downscaled projections for the selected periods were retrieved from the CHELSA v2.1 database (Karger et al., 2017; https://chelsa-climate.org/cmip6/), maintaining spatial resolution consistency.

All environmental layers were clipped to the study area and converted to ASCII format for model compatibility. To reduce multicollinearity, a Pearson correlation analysis was performed on the 20 selected environmental layers. Variables with correlation coefficients ≥ ± 0.85 were eliminated (Elith et al., 2010; Stohlgren et al., 2010). In deciding which variables to retain, we first excluded several bioclimatic layers (Bio9, 18 and 19) due to documented spatial artifacts (Ribeiro et al., 2017) and then we prioritized biologically meaningful extremes (e.g. minimum or maximum values) over mean conditions.

### 2.5 Baseline MaxEnt modeling

Baseline species distribution models were developed using MaxEnt v.3.4.4 (Phillips et al., 2006). Models were run using 15 cross-validated replicates, with 70% of the data randomly partitioned for training and the remaining 30% for testing. The complementary log-log (Cloglog) output format was selected, as it provides an interpretable estimate of occurrence probability under presence-only modeling assumptions. The output consists of a raster map with values ranging from 0 to 1, representing the likelihood of species presence.

Model settings included 10,000 background points, a maximum of 1,000 iterations and a convergence threshold of 0.00001. Model performance was evaluated using the Area Under the Receiver Operating Characteristic Curve (AUC), averaged across replicates. The AUC value indicates how effectively the model distinguishes between presence records and random background points. AUC values typically range from 0.5 to 1, with values closer to 1 indicating stronger relationships between the environmental variables and the predicted species distribution, thus suggesting better model performance. Models with AUC > 0.75 were considered to have satisfactory discriminatory power. Variable importance was assessed through percent contribution, permutation importance, and Jackknife analyses, quantifying the unique explanatory value of each predictor.

Variable importance was assessed through complementary approaches: i) *percent contribution* tracking the increase in regularized gain during model training, ii) *permutation importance* measuring the decrease in AUC when variables were randomly permuted; iii) *Jackknife test*, quantifying the unique explanatory value of each predictor. Specifically for the permutation importance, the random rearrangement tests how model performance changes when the relationship between a variable and species presence is disrupted.

### 2.6 Model calibration and transferability assessment using *kuenm*

To explicitly address concerns related to overfitting, model complexity and transferability to novel climatic conditions, a second modeling framework was implemented using the *kuenm* R package (Cobos et al., 2019). Presence records were randomly partitioned into training (70%) and testing (30%) subsets using kuenm occsplit, ensuring consistency with the baseline MaxEnt setup.

Environmental predictors were combined into candidate variable sets using kuenm varcomb, generating 37 subsets with a minimum of six variables each. An exhaustive calibration was performed using kuenm_cal, generating 666 candidate models by combining six regularization multipliers (0.1, 0.25, 0.5, 1.0, 2, 4), three feature class combinations (lq, lqp, q) and the 37 variable sets.

Candidate models were evaluated using partial receiver operating characteristics (ROC) tests, omission rates at a 5% threshold, and Akaike Information Criterion corrected for small sample sizes (AICc). Model selection followed the “OR_AICc” criterion, retaining only statistically significant models with omission rates ≤ 0.05 and minimum AICc values.

The selected model configuration was subsequently replicated ten times using cross-validation and projected to both current and future climatic scenarios using kuenm mod, choosing the extrapolation and clamping (EC) option for model transfer. Projections were generated in cloglog format, and jackknife analyses were enabled to quantify the unique contribution of individual predictors.

### 2.7 Extrapolation risk analysis (mobility-oriented parity)

To assess the reliability of model projections under future climatic conditions, we evaluated extrapolation risks using the Mobility-Oriented Parity (MOP) metric (Owens et al., 2013), implemented through the *mop* R package (Cobos et al., 2024). MOP analysis allows identification of areas where projected environmental conditions fall outside the range of those used for model calibration, highlighting regions of strict climatic novelty where model predictions should be interpreted with caution. However, MOP diagnostics address environmental novelty within a given climate model and do not capture uncertainty associated with inter-model variability among different Global Climate Models. Consequently, future suitability maps should be interpreted as relative indications of potential shifts rather than deterministic projections of absolute suitability boundaries.

The analysis was conducted by comparing the multivariate environmental space of the calibration area (M), defined by the selected set of bioclimatic and topographic variables used to train the model (Set_28, see Results), with future climatic conditions under the most extreme emission pathways (SSP5-8.5 for 2071-2100). We implemented the basic MOP formulation, which identifies grid cells in the projection area exhibiting at least one environmental variable outside the calibration range. Distance-based MOP variants were not calculated, as the primary aim was to delineate areas of non-analogous climatic conditions rather than to quantify degrees of environmental dissimilarity. Resulting MOP raster classifies areas with strict extrapolation (non-analogous conditions) versus areas within the environmental domain of model calibration, supporting a transparent interpretation of future suitability projections and their associated uncertainty.

### 2.8 Thresholding and suitability classification

The cloglog Maxent model output provides an estimate of species presence probability on a scale from 0 to 1 (Phillips, 2017). However, ecological applications often require a binary classification, such as presence or absence. While this discretization simplifies interpretation, it inevitably results in some loss of information. Despite its conceptual simplicity, determining an appropriate threshold for this conversion is not a straightforward task. Various methods exist, broadly classified as subjective or objective approaches. Subjective thresholds should always be avoided, as they rely on arbitrary cutoffs without any ecological basis. For a comparative analysis of commonly used thresholding methods, see Liu et al. (2005).

Specifically, only for the *post-hoc* analyses related to the soil and aridity factors, we adopted one of the most reliable objective methods: maximizing the sum of training sensitivity and specificity (Liu et al., 2016). This approach identifies the point on the ROC curve where the tangent slope equals 1, making it particularly well-suited for models using pseudo-absence data, like MaxEnt. Sensitivity (or recall) measures the proportion of actual presences correctly identified by the model, while specificity quantifies the proportion of actual absences correctly classified. This balance ensures an optimal trade-off between omission and commission errors, enhancing the model’s ecological relevance.

### 2.9 *Post-hoc* analyses: soil and aridity factors

Soil and aridity variables were analyzed *post-hoc* to explore potential environmental constraints beyond the climatic domain. We gathered comprehensive data on soil suitability for olive cultivation and linked them with available soil spatial information from the World Soil Information Service (WoSIS) database. From the Digital Elevation Model (DEM) we derived slope and slope aspect for the study area. Given the complexity of implementing these parameters in the original model, we opted to address them in post-processing analysis, translating various physical and chemical soil limitation information into their respective soil types according to the Reference Soil Group (RSG) of the World Reference Base for Soil Resources (IUSS Working Group WRB, 2022).

We compared the MaxEnt reclassified output (suitable vs unsuitable) with a possible proxy for olive suitability, the Aridity Index AI (UNEP, 1992; Equation 1), defined as:

(1) $$Aridity\;index=\;\frac{R_{a}}{ET_{0}}$$

Where R_a_ (mm) is the yearly rainfall and ET_0_ (mm) is the yearly reference evapotranspiration (Allen et al., 1998). The AI spatial dataset for 1) actual (WorldClim v.2.1 1970-2000), 2) ssp 126 2041-2060 (multimodel ensemble) and 3) ssp 585 2041-2060 (multimodel ensemble) was retrieved from Zomer et al. (2025). All the rasters were reprojected into EPSG: 3035 - ETRS89-extended/LAEA Europe. Interquartile ranges (Q25-Q75) of aridity index values in suitable areas were calculated to establish potential threshold ranges for olive suitability. The aridity index or the topographic variables, which indirectly incorporate several bioclimatic/topographic variables used in the model, could have introduced multicollinearity issues; therefore, we opted to address this through a *post-hoc* analysis.

### 2.10 Software

Spatial data processing and visualization were performed using QGIS LTR 3.34.7 (QGIS.org, 2025), SAGA GIS 9.6.2 (Conrad et al., 2015 ) and ArcGIS Pro 3.4.2 (Esri, 2025). Species distribution modeling was implemented through MaxEnt 3.3.4 (Phillips et al., 2006; https://biodiversityinformatics.amnh.org/open_source/maxent/) and the *kuenm* package (Cobos et al., 2019) in R, while statistical analysis was performed in R v. 4.4.0 (R Core Team, 2024) using the terra package (Hijmans, 2025) for raster processing and dplyr (Wickham et al., 2023), ggplot2 (Wickham, 2016), and patchwork (Pedersen, 2025) for data manipulation and visualization. The mop package (Cobos et al., 2025) was employed for MOP analysis. Microsoft Excel was employed for data organization and preliminary analyses.

## 3 Results

### 3.1 Environmental predictor selection and model calibration

**Figure 3:**
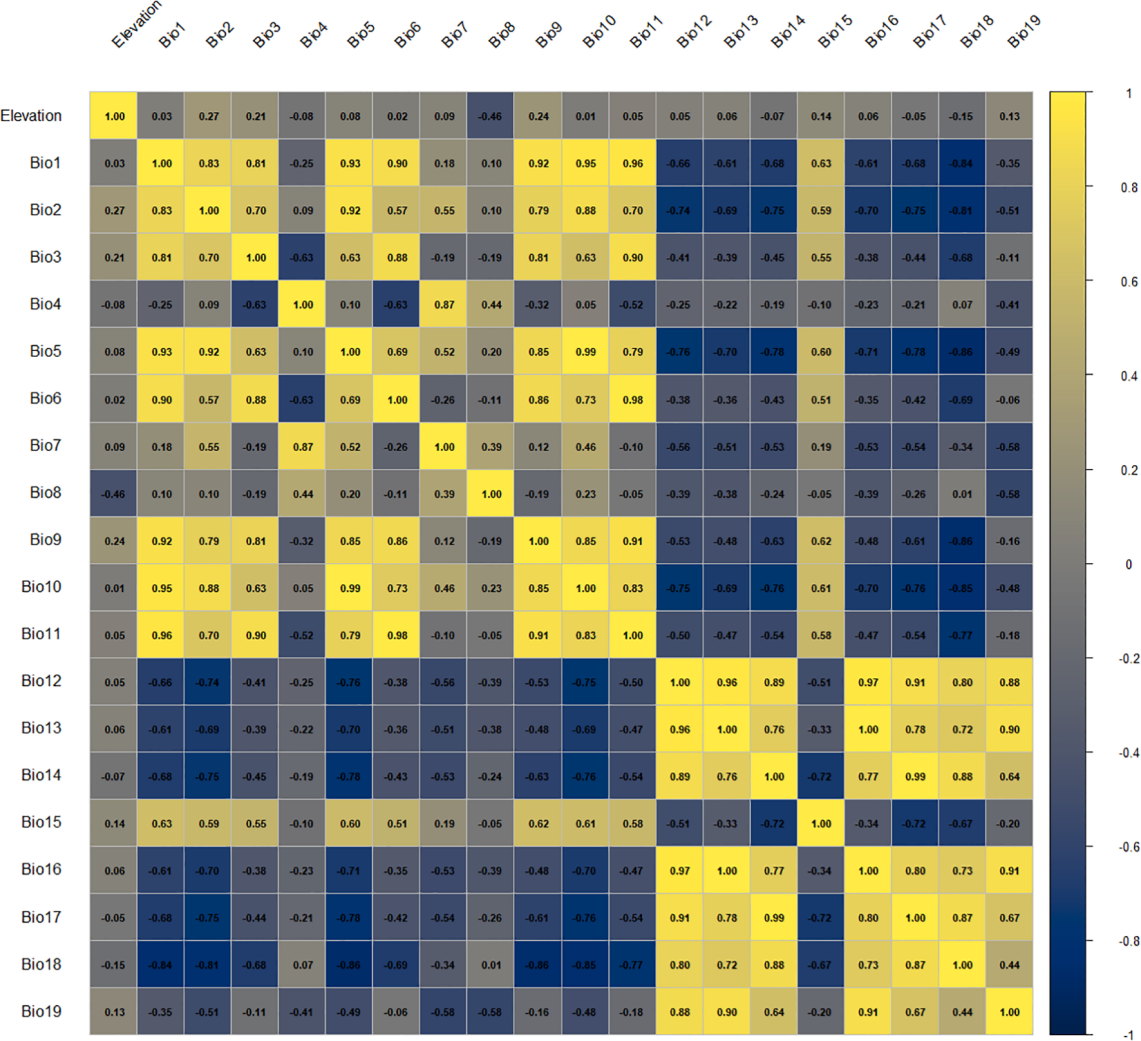
Pearson correlation matrix of the 20 initial environmental predictors.

The Pearson correlation analysis applied to the initial set of 20 environmental predictors (**Figure 3**) resulted in a final subset of eight environmental variables, representing key temperature- and precipitation-related gradients together with elevation (**Table 2**).

**Table 2:** Bioclimatic variables retained for MaxEnt modeling after correlation filtering. Includes variable codes, definitions, and units.

Code	Bioclimatic variables	Unit
Elevation	Elevation	Meters
Bio4	Temperature Seasonality (standard deviation ×100)	Celsius degree
Bio5	Max Temperature of Warmest Month	Celsius degree
Bio6	Min Temperature of Coldest Month	Celsius degree
Bio8	Mean Temperature of Wettest Quarter	Celsius degree
Bio13	Precipitation of Wettest Month	Millimeters
Bio14	Precipitation of Driest Month	Millimeters
Bio15	Precipitation Seasonality (Coefficient of Variation)	Millimeters

Two modeling approaches were implemented. First, a baseline MaxEnt model was calibrated using commonly adopted default settings and 15-fold-cross validation, representing a standard correlative SDM framework. Second, a rigorously calibrated model was developed using the *kuenm* package, which enables systematic exploration of model complexity through a combination of feature classes, regularization multipliers, and alternative variable subsets. Model calibration using the *kuenm* framework involved the evaluation of a total of 666 candidate models, generated from all possible combinations of six regularization multiplier values, three feature class sets and 37 alternative subsets of environmental predictors. Candidate models were evaluated following a hierarchical selection procedure based on statistical significance (partial ROC), omission rates (OR ≤ 5%), and model parsimony (AICc).

All candidate models were statistically significant according to partial ROC tests (full results in SI), only six models satisfied the omission rate criterion, and only one model simultaneously met all selection criteria, including minimum AICc. The selected model (M_025_F_lqp_Set_28) was characterized by a regularization multiplier of 0.25, a linear-quadratic-product (lqp) feature class combination and a reduced set of predictors (Set 28, without the precipitation-related predictors Bio13 and Bio14). This model showed an omission rate of 0.049, satisfying the predefined threshold, and the lowest AICc value, with ΔAICc = 0 and AICc weight = 1.

Compared to the baseline model (**Table 3**), which was strongly dominated by the minimum temperature of the coldest month (Bio6), the *kuenm*-optimized model exhibited also a more even contribution among predictors, including temperature seasonality (Bio4), maximum temperature of the warmest month (Bio5), mean temperature of the wettest quarter (Bio8), and precipitation seasonality (Bio15). These differences highlight the sensitivity of variable importance estimates to calibration strategy rather than fundamental changes in species-environmental relationships.

**Table 3:** Relative importance of each bioclimatic variable based on percent contribution and permutation importance across the model replicates.

Variable	Percent contribution (%)			Permutation importance (%)		
	Baseline	Kuenm	Δ	Baseline	Kuenm	Δ
Bio4	1.23 ± 0.07	19.77 ± 1.55	18.54	2.62 ± 0.10	14.96 ± 2.58	12.34
Bio5	9.68 ± 0.61	10.36 ± 0.65	0.68	14.51 ± 0.40	37.54 ± 4.89	23.03
Bio6	53.07 ± 0.24	50.95 ± 0.67	-2.12	57.51 ± 0.39	28.37 ± 6.25	-29.14
Bio8	0.22 ± 0.04	11.53 ± 1.09	11.31	0.98 ± 0.09	8.35 ± 1.45	7.37
Bio13	23.47 ± 0.07	–	-23.47	19.5 ± 0.31	–	-19.5
Bio14	1.17 ± 0.09	–	-1.17	2.83 ± 0.08	–	-2.83
Bio15	11.13 ± 0.65	6.83 ± 0.58	-4.3	1.79 ± 0.06	5.96 ± 1.55	4.17
Elevation	0.03 ± 0.01	0.56 ± 0.79	0.53	0.27 ± 0.04	4.81 ± 0.83	4.54
	Baseline			Kuenm		
Training AUC	0.78 ± 0.00			0.77 ± 0.00		
Test AUC	0.78 ± 0.00			0.77 ± 0.01		

### 3.2 Model performance and transferability assessment

**Figure 4:**
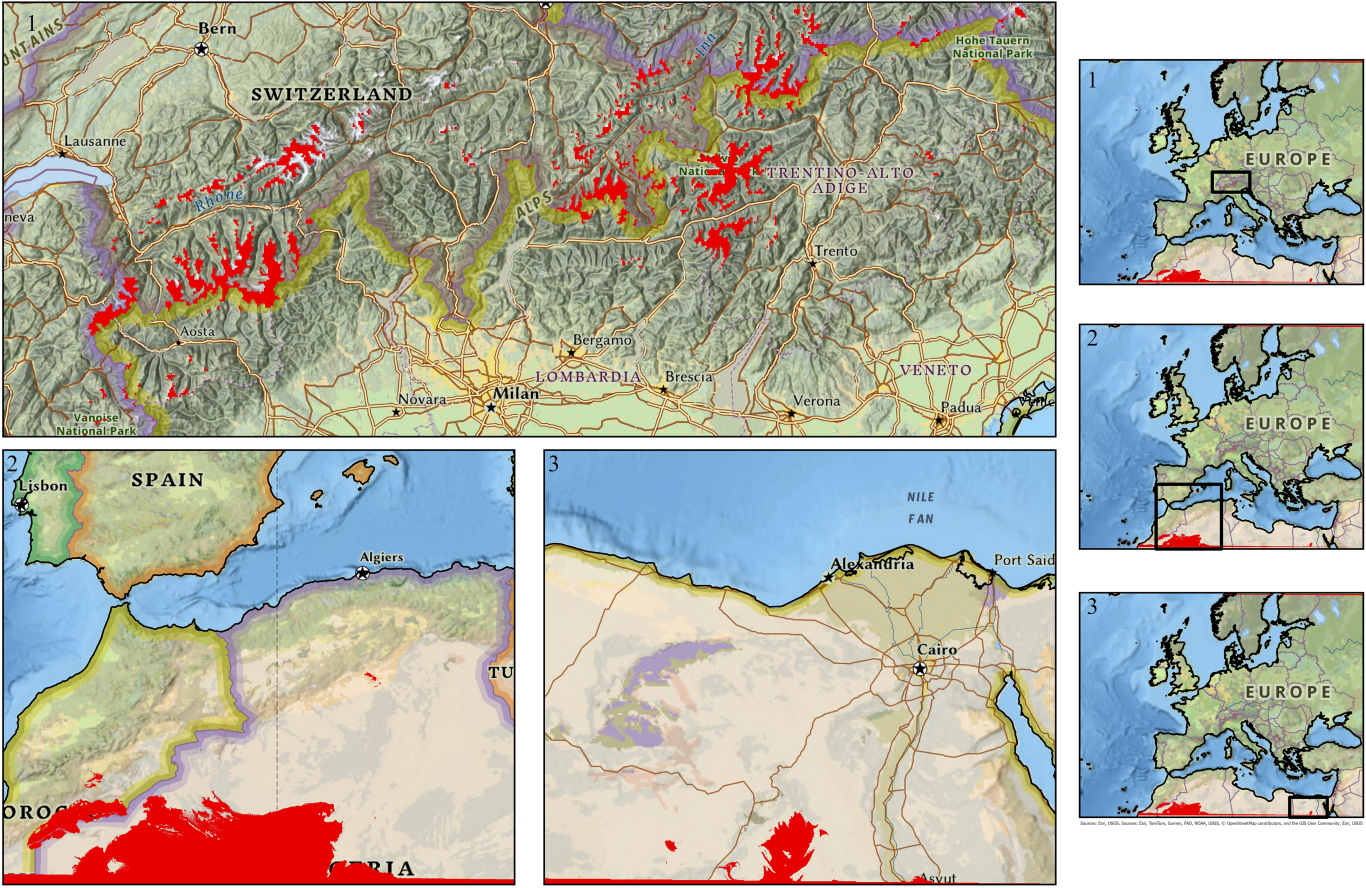
Mobility-oriented parity (MOP) analysis for the study area.

Both modeling approaches achieved comparable discriminatory performance (**Table 3**). The baseline MaxEnt model yielded a mean test AUC of 0.78 (± 0.00), while the kuenm-optimized model achieved a mean test AUC of 0.77 (± 0.01). Training and test AUC values were closely aligned in both cases, suggesting no strong evidence of overfitting at the scale of the analysis, while acknowledging that spatial autocorrelation may still influence evaluation metrics. The full set of results, together with ROC curves and Jackknife tests, can be found in SI. To explicitly evaluate model transferability under future climate scenarios, Mobility-Oriented Parity (MOP) analyses were conducted for the most extreme emissions pathway (SSP5-8.5, 2071-2100). MOP results (**Figure 4**) identified areas characterized by strict extrapolation, where future climatic conditions fall outside the environmental domain used for model calibration, primarily in high-altitude Alpine areas and in hyper-arid regions of Sahara and southern Egypt: projected suitability in these areas should therefore be interpreted as highly uncertain rather than as reliable predictions.

### 3.3 Current and future climatic suitability patterns

**Figure 5:**
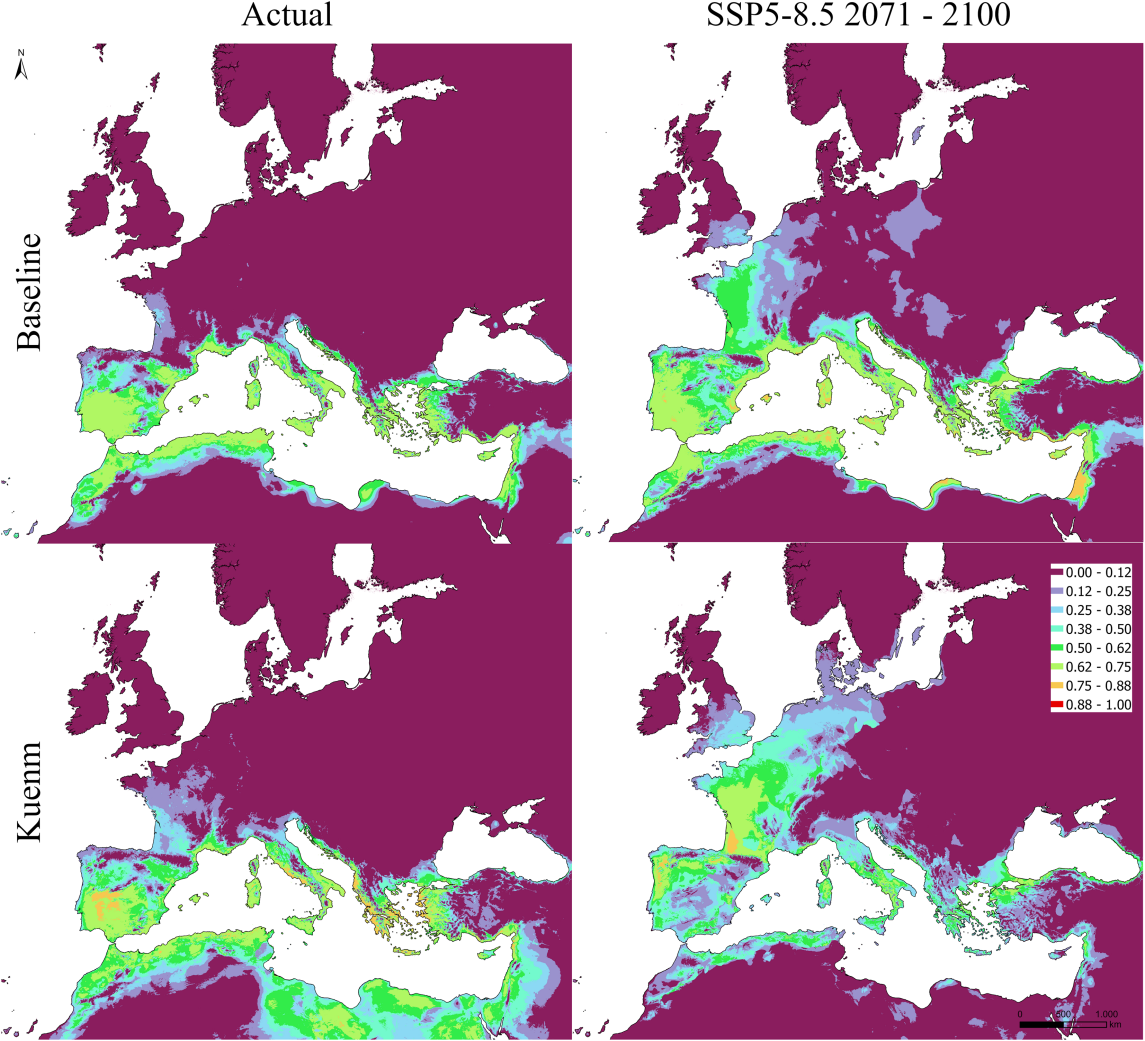
Projected suitability maps using MaxEnt, raw output. Top panels show the baseline model, while the bottom ones the kuenm-based framework. Left = actual period; right = SSP5-8.5 scenario 2071-2100.

**Figure 6:**
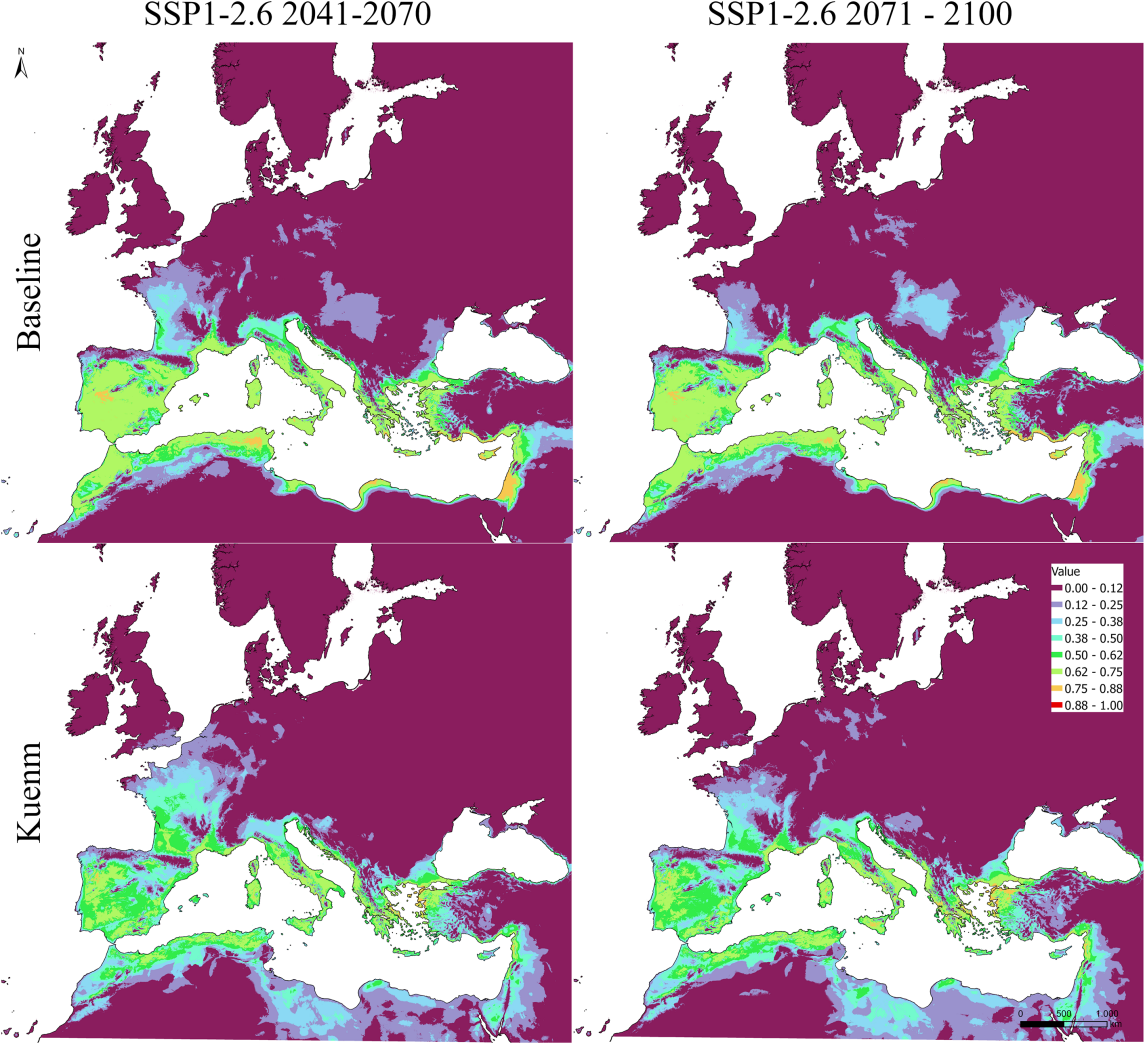
Projected suitability maps using MaxEnt, raw output. Top panels show the baseline model, while the bottom ones the kuenm-based framework. Left = SSP1-2.6 scenario 2041-2070; right = SSP1-2.6 scenario 2071-2100).

**Figure 7:**
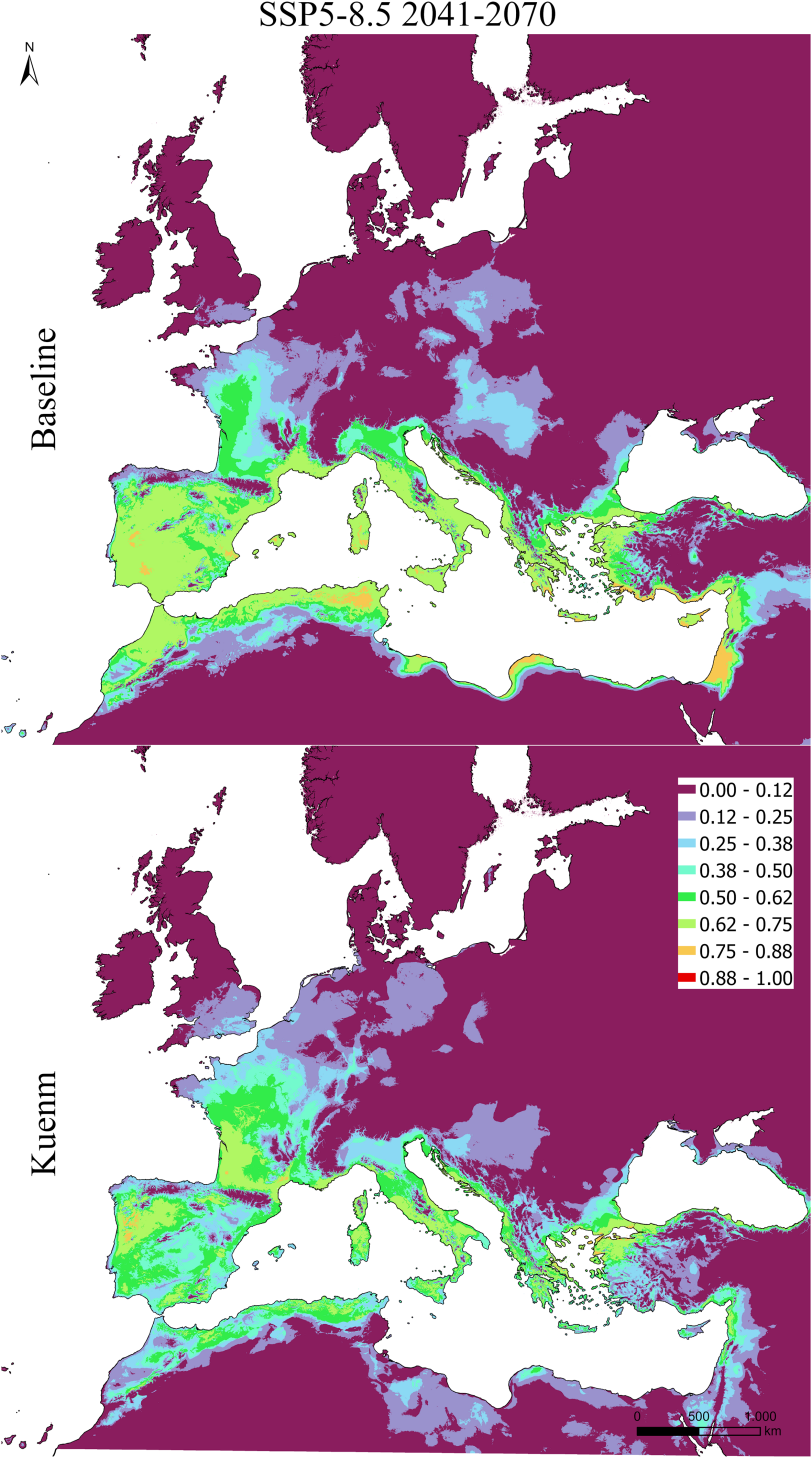
Projected suitability maps using MaxEnt, raw output. Top panel show the baseline model, while the bottom one the kuenm-based framework, for SSP5-8.5 scenario 2041-2070.

Under current climate conditions, both the baseline and *kuenm*-optimized models reproduced the broad spatial distribution of olive cultivation across the Mediterranean Basin, with high suitability concentrated in southern Europe, coastal North Africa, and the eastern Mediterranean (**Figures 5**–**7**). Future projections suggested a broadly consistent large-scale trend across modeling approaches. Under the low-emission scenario (SSP1-2.6), changes in climatic suitability were moderate and largely confined to marginal areas, with the persistence of a core Mediterranean suitability zone. Under the high-emission scenario (SSP5-8.5), projections were associated with a more pronounced redistribution, including potential contraction in parts of southern and eastern Mediterranean regions and potential expansion toward higher latitudes, particularly in France, northern Italy, and the northern Europe.

### 3.4 Soil limiting factors and soil type

Based on recommendations on soil physical, chemical and topographic parameters in Famiani et al., 2023, we identified several soil types (Reference Soil Group RSG) classified under the WRB as potentially poorly suited for olive cultivation (**Table 4**; **Supplementary Figure 5**). These include Leptosols, due to their shallow depth and limited root development potential; Vertisols, which are prone to waterlogging and physical instability caused by high clay content; Stagnosols and Gleysols, which are associated with poor drainage and anaerobic conditions. Additionally, Solonetz and Solonchaks often exhibit excessive levels of exchangeable sodium and salinity, respectively, beyond the tolerance thresholds of olive trees. Histosols, due to their high organic matter, acidity, and poor structural stability, also present unfavorable. In rare cases, strongly acidic Andosols may limit nutrient availability and increase toxic element solubility. These soil types were mapped and are available in SI. Although some soil types present clear limitations for olive cultivation, some of these constraints – such as acidity, poor structure, or excess salinity – can be effectively managed through targeted agronomic practices, including the application of physiologically basic amendments and appropriate drainage or soil conditioning measures. This highlights the importance of not only identifying limiting factors, but also evaluating their potential for correction within a sustainable management framework. Lastly, two topographic maps were produced: slope aspect (**Supplementary Figures 7**–**S8**), to identify the best location in area at frost risk (south), and slope (**Supplementary Figure 6**), to identify the best area for olive mechanization (slope ≤ 15-30%).

**Table 4:** Link between soil constraints and reference soil groups (World Reference Base 2022). Physical and chemical limitations for olive cultivation and soil types associated with these constraints.

Limitation	Value	Potential poorly suitable soil type
Soil depth	At least 50 cm	Leptosols
Water logging, clay content	40%	Vertisols, Gleysols, Stagnosols
Salinity, electrical conductivity	8 dS/m	Solonetz, Solonchaks
Sodium, Exchangeable Sodium Percentage	20-40%	
pH	> 5.5 and < 8.5	Podzols

### 3.5 Aridity Index and olive suitability

Statistical comparisons of aridity index distributions between suitable and unsuitable areas, based on the baseline MaxEnt model, revealed highly significant differences across all scenarios. For exploratory and *post-hoc* purposes, continuous probability values were converted into a binary representation of habitat suitability. The defined threshold was set to 0.3472, corresponding to the cloglog value at which the sum of training sensitivity and specificity is maximized. The two-sample t-test analysis yielded extremely strong statistical significance (p < 0.001) for all scenarios, reflecting the large sample sizes and substantial differences in aridity patterns between suitability classes. Descriptive statistics are shown in SI.

## 4 Discussion

### 4.1 Advances beyond previous basin-scale assessments

Our results suggest a potential northward shift in climatically suitable areas for olive cultivation, particularly in France and northern Europe, while traditional regions in southern Europe and North Africa retain overall suitability but may face growing stress from heat and aridity. This spatial reorganization highlights both potential opportunities and emerging challenges for Mediterranean olive systems under climate change. Newly suitable areas, currently marginal or unexploited, may become climatically suitable for high-density, mechanized orchards, especially in flat regions, following cultivation models already adopted in southern Spain, Italy, Portugal, California, and Australia. However, agronomic viability cannot be inferred directly from climatic suitability alone. These new areas face constraints such as cold extremes, limited infrastructure, lack of local expertise, and risks of pathogen introduction, including the quarantine bacterium *Xylella fastidiosa*.

Previous studies support these considerations. For example, Tóth and Végvári (2016) applied MaxEnt to predict future winegrape regions in Europe, showing that crops with higher plasticity than olive may expand more extensively. Similarly, Bosso et al. (2016a) modelled the potential distribution of *X. fastidiosa* in the Mediterranean region using MaxEnt, identifying precipitation during the driest (30.7%) and wettest (30.3%) months as the main factors shaping the bacterium’s distribution. Notably, France currently appears free from this pathogen, making it a particularly interesting target for potential expansion.

Previous assessments of olive climatic suitability across the Mediterranean have relied on a range of modeling approaches that differ substantially in scope, resolution, and conceptual framing. For example, Moriondo et al. (2008) compared different statistical classifiers across the Mediterranean Basin using coarser-resolution climatic data, while Ponti et al. (2014) developed a basin-scale, physiologically based demographic model explicitly describing olive and olive fly biology, and Ponti et al. (2024) applied a similar mechanistic framework at sub-national scale (Andalusia), using multi-model climate projections.

The present study combines basin-wide coverage with high-resolution climatic predictors (30 arc-second resolution) and integrates rigorous model calibration and explicit transferability diagnostics. This approach bridges the gap between coarse-resolution basin-scale SDMs and highly detailed regional process-based models, providing a spatially explicit and methodologically robust assessment of potential climatic suitability shifts across the Mediterranean under future climate change.

### 4.2 Model calibration, complexity and transferability

**Figure 8:**
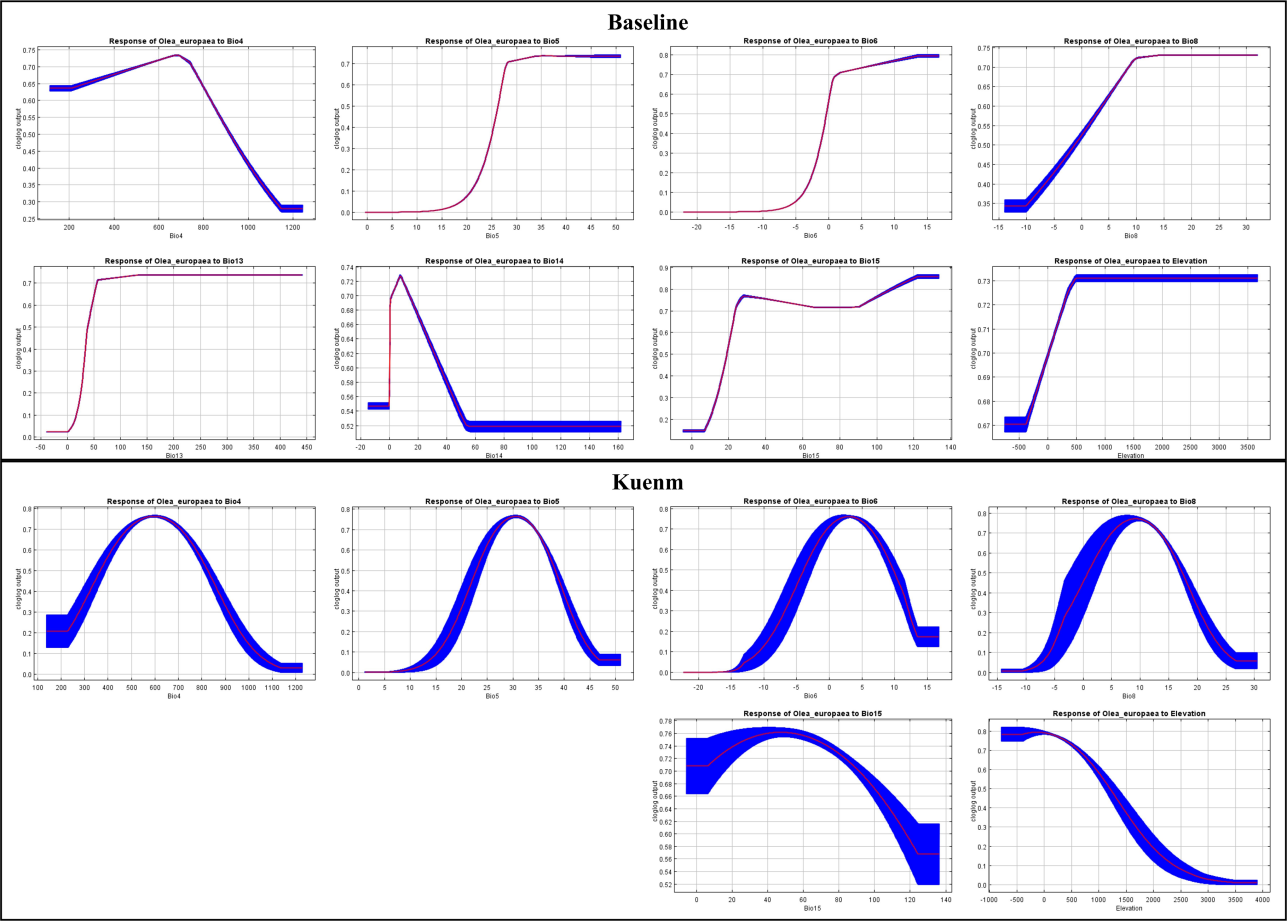
Response curves for the environmental variables, for the baseline model (top) and the kuenm-framework (bottom). Curves show how the predicted probability of olive presence changes with each variable, holding others constant. Red line: mean response; shaded blue area: standard deviation across replicates.

A central contribution of this study lies in the explicit comparison between a baseline MaxEnt model and a *kuenm*-optimized configuration. Rather than treating these as competing models, they should be interpreted as complementary representations of the same correlative framework under different calibration philosophies. Response curves derived from the baseline MaxEnt model frequently exhibited narrow optima, abrupt thresholds, and irregular shapes across several climatic predictors, particularly temperature seasonality (Bio4), extreme temperature variables (Bio5, Bio6), and precipitation seasonality (Bio15). Such patterns are indicative of high model sensitivity to specific environmental ranges and suggest potential overfitting, especially in the context of projections to future climate scenarios (**Figure 8**).

In contrast, response curves obtained from the *kuenm*-optimized model were consistently smoother and more unimodal, with broader tolerance ranges and gradual transitions at environmental extremes. This behavior reflects the effect of explicit model complexity control through regularization tuning and variable set selection. Importantly, differences in response curve shape indicate that parameter tuning mainly affected model complexity rather than the direction of species–environment relationships, thereby increasing confidence in the relative robustness of projections under future climate scenarios.

### 4.3 Interpretation of predictors: correlations rather than mechanisms

The spatial pattern of climatic suitability largely reflects the physiological limitations of the olive tree. Historically, olive cultivation has been concentrated within the latitudinal range of 30° to 45°, a distribution significantly influenced by temperature constraints (Petruccelli et al., 2022). This distribution is also shaped by its physiological responses to water and heat stress. Gucci and Sebastiani (2023) emphasize the presence of structural and metabolic adaptations – such as reduced stomatal conductance, thickened leaf cuticles, and the accumulation of osmoprotective compounds – that enhance tolerance to drought, salinity, and high temperatures. Sebastiani (2011) further highlights key physiological mechanisms under water deficit, including stomatal closure, osmotic regulation, and the synthesis of stress-related metabolites, all of which help maintain photosynthetic efficiency and hydraulic function under prolonged dry conditions.

The olive tree can tolerate brief exposure to sub-zero temperatures via supercooling mechanisms (Fiorino and Mancuso, 2000), but prolonged frost events below -8°C remain critical thresholds for survival and productivity (Palliotti and Bongi, 1996; Vitagliano and Sebastiani, 2002). The strong influence of Bio6 (53.07% contribution in the Baseline and 50.95% in the *kuenm* framework) in our models is consistent with broad thermic constraints that limit northward occurrence patterns. Conversely, warmer winters may jeopardize productivity in traditional regions by undermining (Ayerza and Sibbett, 2001), as accumulated exposure to cold temperatures enables the plants to set up inflorescence production when warmer temperatures occur properly. Recent research from subtropical climates (e.g., Medina-Alonso et al., 2023) documents irregular budburst and asynchronous flowering under insufficient winter chilling, raising concerns for olive cultivation in already warm areas such as North Africa, South Italy, and Andalusia (Spain) under future warming.

Interpretation of variable importance and response curves is explicitly framed within the correlative nature of MaxEnt. While the minimum temperature of the coldest month (Bio6) consistently emerged as the dominant predictor, this reflects a strong statistical association rather than a direct physiological mechanism.

The importance of Bio6 likely captures broad thermic constraints shaping the realized climatic niche of olive cultivation across the Mediterranean, rather than specific cold-tolerance thresholds. Similarly, the contribution of precipitation-related variables such as Bio13 in the baseline model should be interpreted as a proxy for overall moisture regimes and correlated seasonal dynamics, rather than as evidence of a mechanistic influence of wettest-month precipitation on olive suitability.

This distinction is particularly important given that olive phenology and productivity are governed by complex interactions between temperature, water availability, cultivar-specific traits, and management practices. Consequently, while the observed correlations are ecologically meaningful at the basin scale, they should not be interpreted as causal physiological responses.

While precipitation of the wettest month (Bio13) ranked as the second most influential variable only in the baseline MaxEnt model (23.47% contribution; 19.5% permutation importance), its ecological interpretation must be treated with caution. In most traditional olive-growing areas, the wettest month typically occurs during winter, outside critical phenological phases such as flowering and fruit set. Therefore, the high importance of Bio13 in the baseline model reflects its correlation with broader moisture regime rather than a direct mechanistic control on olive climatic suitability. This interpretation is supported by the *kuenm*-optimized model, in which Bio13 (and Bio14) were excluded during calibration without loss of predictive performance (test AUC: 0.77 vs 0.78 in the baseline). This contrast indicates that the apparent importance of Bio12 in the baseline configuration partly results from sensitivity to correlated predictors rather than from robust basin-scale climatic constraints.

Nevertheless, precipitation remains ecologically relevant for olive systems, primarily as a regulator of productivity. This distinction aligns with the framework proposed by Moriondo et al. (2013), in which temperature defines the fundamental niche while precipitation constrains the realized niche, and with previous Mediterranean-wide studies highlighting temperature and rainfall as dominant predictors of olive distribution (Moriondo et al., 2008; Kassout et al., 2022).

In newly suitable northern areas, future changes in seasonal precipitation regimes may still represent a critical limiting factor. If the wettest month shifts toward spring or early summer, increased rainfall during flowering could elevate the risk of pollen washout and reduce fruit set, although this remains speculative without higher-resolution phenological and seasonal rainfall data. Similar concerns have been raised in recent modeling studies for wild olive populations in Morocco and Türkiye, where precipitation and temperature jointly drive biogeographical dynamics (Kassout et al., 2022; Çıvğa, 2024).

Notably, our projections highlight an emerging climatic tension across the Mediterranean Basin. Northern expansion zones gain thermic suitability but may experience suboptimal precipitation regimes characterized by three potential mismatches: (1) spring rainfall misaligned with flowering phenology, increasing waterlogging and pollen washout risks; (2) shortened growing seasons that compress fruit maturation; and (3) intensified autumn rainfall disrupting harvest operations and favouring fungal pathogens such as *Colletotrichum* spp. Conversely, traditional olive-growing regions in southern Europe face increasingly severe heat–water stress combinations, threatening long-term economic sustainability despite maintained climatic suitability. Empirical evidence from Italy indicates that warming and increasing drought have facilitated olive expansion into previously marginal northern regions, while southern areas are increasingly constrained by water scarcity (Rodrigo-Comino et al., 2021).

Precipitation seasonality (Bio15) exhibited a moderate contribution in the baseline model (11.13%), but its relatively low permutation importance (1.79%) suggests partial redundancy with other variables. In the optimized model, Bio15 retained a smaller but more stable role (6.83% contribution; 5.96% permutation importance), supporting its interpretation as a secondary modifier rather than a primary climatic constraint. In contrast, temperature-related predictors remained consistently influential across both modeling approaches. Minimum temperature of the coldest month (Bio6) dominated both models, although its permutation importance decreased substantially in the optimized model (from 57.5% to 28.4%), reflecting a redistribution of explanatory power among correlated thermal variables rather than a reduced ecological relevance.

The maximum temperature of the warmest month (Bio5) showed stable contributions across both models and a marked increase in permutation importance in the optimized model (from 14.5% to 37.5%), highlighting the role of heat stress in constraining olive suitability at southern latitudes. Experimental studies confirm that extreme temperatures during flowering can reduce pollen viability and accelerate floral desiccation, ultimately impairing yield (Benlloch-González et al., 2019). The increased importance of temperature seasonality (Bio4) and mean temperature of the wettest quarter (Bio8) in the optimized model further suggests that thermal variability, rather than isolated extremes or single precipitation metrics, plays a critical role in shaping basin-scale olive suitability.

Overall, the prevalence of temperature-related variables, accounting for approximately two-thirds of the model explanatory power, aligns with previous findings indicating that thermal thresholds define the fundamental niche of Mediterranean perennial crops, while precipitation primarily regulates productivity within that niche (Moriondo et al., 2013; Ponti et al., 2014). The negligible contribution of elevation reflects the basin-wide scale of the analysis; although elevation-driven microclimatic effects are relevant locally, they are not primary determinants at this spatial resolution. At finer scales, however, elevation gradients may still offer adaptive opportunities by buffering heat stress and extending cultivation into higher altitudes.

### 4.4 Scale, sampling bias, and modeling strategy

Given the basin-wide scope of this study, the primary objective was to characterize broad climatic constraints on olive cultivation rather than local suitability patterns. At this spatial scale, preserving the full climatic envelope of olive cultivation was prioritized in order to capture the full range of environmental conditions under which olives are currently cultivated across the Mediterranean Basin. Consequently, no spatial thinning or explicit correction for sampling bias was applied. Occurrence data derived from CLC2018 and GBIF are spatially clustered and reflect heterogeneous sampling effort, particularly in intensively cultivated and easily accessible regions. Sampling bias was therefore acknowledged but not quantitatively assessed or corrected, and residual bias may still influence model calibration, variable importance, and evaluation metrics. For this reason, AUC values and omission rates should be interpreted cautiously, as spatial autocorrelation and clustered presence records may inflate apparent model performance. Rather than attempting to correct sampling bias directly, we adopted an indirect strategy focused on controlling model complexity and transferability. Specifically, we implemented a rigorous calibration framework (*kuenm*) that explicitly balances model fit and complexity using omission rates and AICc, and explicitly evaluated extrapolation risk using MOP analyses.

Several studies have demonstrated that correcting for geographical sampling bias can improve SDM performance (Kramer-Schadt et al., 2013; Syfert et al., 2013). However, such corrections are not universally optimal and may not fully resolve bias effects at continental extents with heterogeneous data sources. Moreover, the effectiveness of bias correction depends on the availability of independent validation data and the spatial structure of occurrence records (Dubos et al., 2022). In this context, model calibration and extrapolation control represent key complementary strategies for improving robustness.

### 4.5 Spatial resolution, uncertainty, and limits of inference

The spatial resolution adopted (~1 km²) is appropriate for basin-scale analyses and allows identification of broad climatic gradients shaping olive suitability across the Mediterranean. However, this resolution does not support fine-scale agronomic recommendations. Local conditions such as cultivar choice, irrigation infrastructure, soil management, and microclimatic variability may substantially modify suitability at the farm or plot level.

Accordingly, projections presented here should be interpreted as probabilistic indicators of regional climatic envelopes rather than precise predictors of future cultivation success. Soil constraints and aridity patterns were therefore explored only in *post-hoc* analyses (Supplementary Information) to contextualize climatic suitability without overstating their predictive role at this scale. Several methodological constraints must be acknowledged to the MaxEnt framework. First, presence-only models are sensitive to sampling bias and spatial autocorrelation (Phillips et al., 2009; Veloz, 2009). The use of CORINE Land Cover and GBIF data ensured broad geographic coverage, but may have overrepresented cultivated and accessible zones, especially in intensively farmed areas, potentially skewing environmental associations. Specifically, clustering can lead to an overrepresentation of environmental conditions associated with high-density areas, potentially inflating the importance of certain predictors while underestimating suitability in less-sampled regions (Dudik et al., 2005; Phillips and Dudík, 2008). Second, the spatial resolution (~1 km²) may mask important microclimatic and edaphic variability at the farm or plot level. Local conditions such as slope exposure, presence of compatible cultivars, or irrigation infrastructure must be considered for olive planning. Third, MaxEnt does not account for species’ physiological plasticity or adaptive capacity. It treats all occurrences as ecologically equivalent, whereas in reality, cultivar-specific responses, local adaptation, and management interventions can significantly influence outcomes. Lastly, the complex behavior of the chilling accumulation in tree crops needs more in study and it is beyond the MaxEnt approach. For instance, the actual chilling accumulation models take into account only the temperature of the air, but new approaches are trying to use the tree temperature to overcome the increasingly variable climate (Guzman-Delgado et al., 2025).

Potential strategies to adopt for the future oliviculture include: i) deficit irrigation (Mairech et al., 2021); ii) selection of drought, heat, and cold-tolerant cultivars; iii) promotion of agrobiodiversity and genetic conservation; iiii) strategic use of south-facing slopes (see slope aspect map in SI). The role of technological innovations, such as CRISPR-edited cultivars or sensor-based irrigation, should not be underestimated. These were not accounted for in our modeling but could substantially alter the future success of olive cultivation, especially in high-risk regions.

## 5 Conclusions

This study provides a basin-wide assessment of present and future climatic suitability for olive cultivation across the Mediterranean, integrating high-resolution climate data with rigorously calibrated species distribution models. Our results confirm that low-temperature constraints primarily define the fundamental climatic envelope of olive cultivation, while precipitation-related variables modulate suitability within thermally permissive regions.

Comparison between baseline and optimized MaxEnt models demonstrates that explicit control of model complexity substantially affects response curve shape and variable importance, without altering the overall direction of species–environment relationships. The optimized modeling framework yielded smoother, more conservative suitability responses and enhanced robustness when projecting under future climate scenarios. Although model variability across replicates and differences between baseline and optimized models provide qualitative insights into model sensitivity, this study does not include formal uncertainty propagation, multi-model climate ensembles, or sensitivity analyses. As a result, uncertainty is assessed qualitatively rather than quantitatively, and future work should explicitly address these aspects.

Projections indicate a progressive northward shift of suitable climates, with emerging opportunities in currently marginal regions and increasing climatic stress in traditional olive-growing areas, particularly under high-emission scenarios. However, suitability gains in northern regions may be constrained by seasonal precipitation regimes and phenological mismatches, while southern regions may retain climatic suitability at the cost of increased water stress and management inputs.

Importantly, explicit evaluation of extrapolation risk revealed that strict non-analogue climatic conditions are spatially limited and largely outside core olive-producing areas, supporting the general transferability of projections while underscoring the need for caution in novel climate domains. While soil characteristics, aridity, and management practices were addressed in a *post-hoc* interpretative framework, future work integrating process-based modeling and cultivar-specific responses will be essential to refine projections at local scales. Overall, this study bridges the gap between coarse basin-scale assessments and detailed regional modeling by providing a spatially explicit, methodologically transparent evaluation of olive climatic suitability under climate change, contributing actionable insights for long-term adaptation strategies in Mediterranean agriculture.

## Data Availability

The datasets presented in this study can be found in online repositories. The names of the repository/repositories and accession number(s) can be found below: https://doi.org/10.5281/zenodo.15721694.
