# Supplementary material for: Projected shifts in climatic suitability of olive (Olea europaea L.) in the Mediterranean
Source: Front Plant Sci. 2026 Mar 13;17:1743577. doi: 10.3389/fpls.2026.1743577 (PMC13021889; doi:10.3389/fpls.2026.1743577)
Supplement: Supplementary file 2 [file Table1.docx]

**Supplementary Information – Projected shifts in climatic suitability of olive (*Olea europaea* L.) in the Mediterranean – Belvisi and Tranchina, 2025.**

A keyword plus analysis reveals how Maxent is predominantly associated with research on climate change and biodiversity, with a focus on modelling the distribution of species and ecosystems (details of the research query on the Scopus database in SI; S2-S3). The prominence in these fields is further underscored by its frequent co-occurrence with terms like 'ecology,' 'habitat,' and 'endangered species.' Furthermore, the analysis reveals a significant interest in applying MaxEnt across diverse geographical regions, including China, the United States, and India, highlighting the global relevance of this tool in ecological and environmental research. Beyond established themes, the tree map also suggests a growing interest in utilizing MaxEnt in emerging areas such as invasive species studies and ecological risk assessment. This comprehensive overview of Maxent’s applications underscores its central role in addressing complex challenges in environmental research. Despite its notoriety, MaxEnt has not yet been applied to predict the suitability of olive trees at the Mediterranean scale, which presents an opportunity for future research to explore the implications of climate change on this important crop for the daily diet of almost one billion people living in the Mediterranean area.


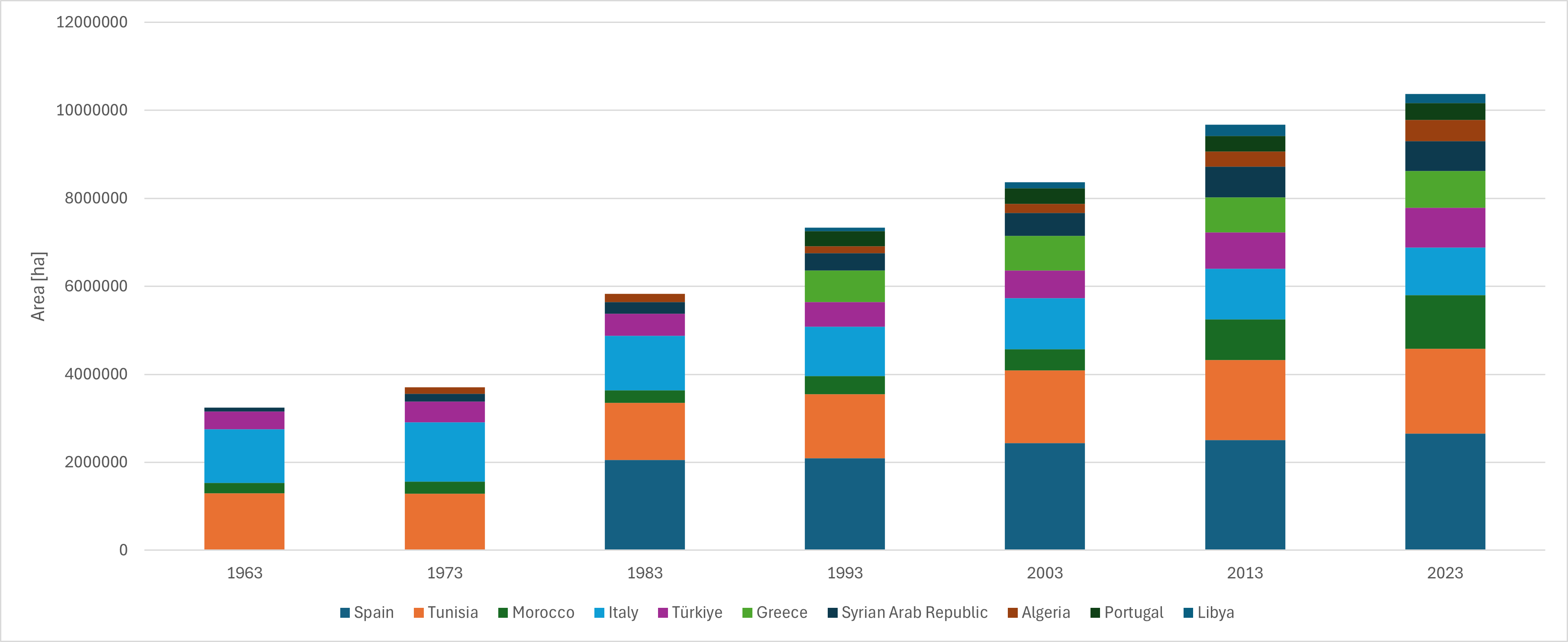


**Figure S1**. *Top 10 Countries by Olive Area Harvested (1963-2023) This bar chart illustrates the historical trends in olive cultivation area (in hectares) for the top 10 producing countries in the Mediterranean region and beyond, from 1963 to 2023. Data was sourced from FAOSTAT (2025)*

**Bibliometric analysis information**

Research query on SCOPUS: (search query: TITLE-ABS-KEY (maxent) AND PUBYEAR > 2005 AND PUBYEAR < 2026 AND (LIMIT-TO (LANGUAGE, "English"), accessed February 8^th^, 2025) with the bibliometrix package (Aria & Cuccurullo, 2017). Total of 7,741 publications, of which 7,629 with complete metadata.


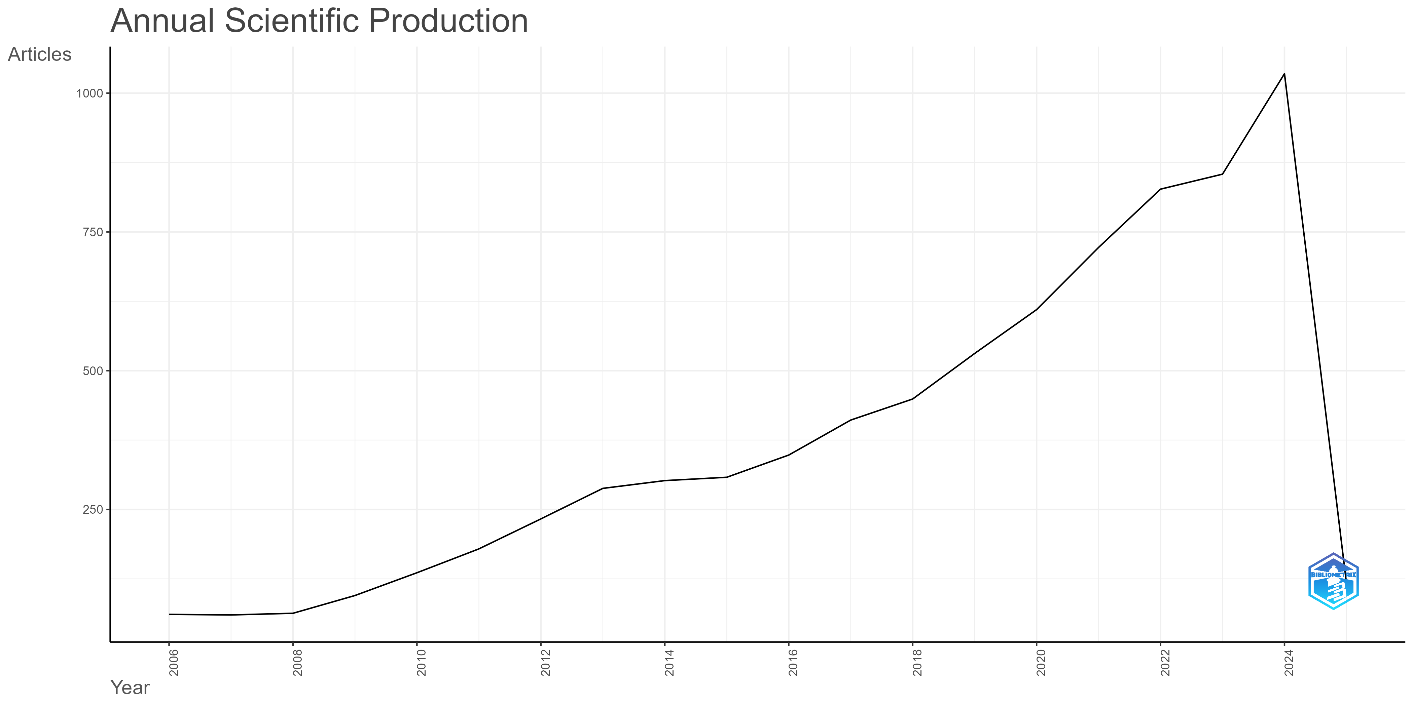


**Figure S2**. *Annual Scientific Production of Maxent Papers (2006-2025) This line graph displays the annual number of scientific articles published on the Maxent model, as indexed in the Scopus database between 2006 and early 2025. The data was obtained through a bibliometric analysis performed on February 8th, 2025, using the 'bibliometrix' package (Aria & Cuccurullo, 2017)*


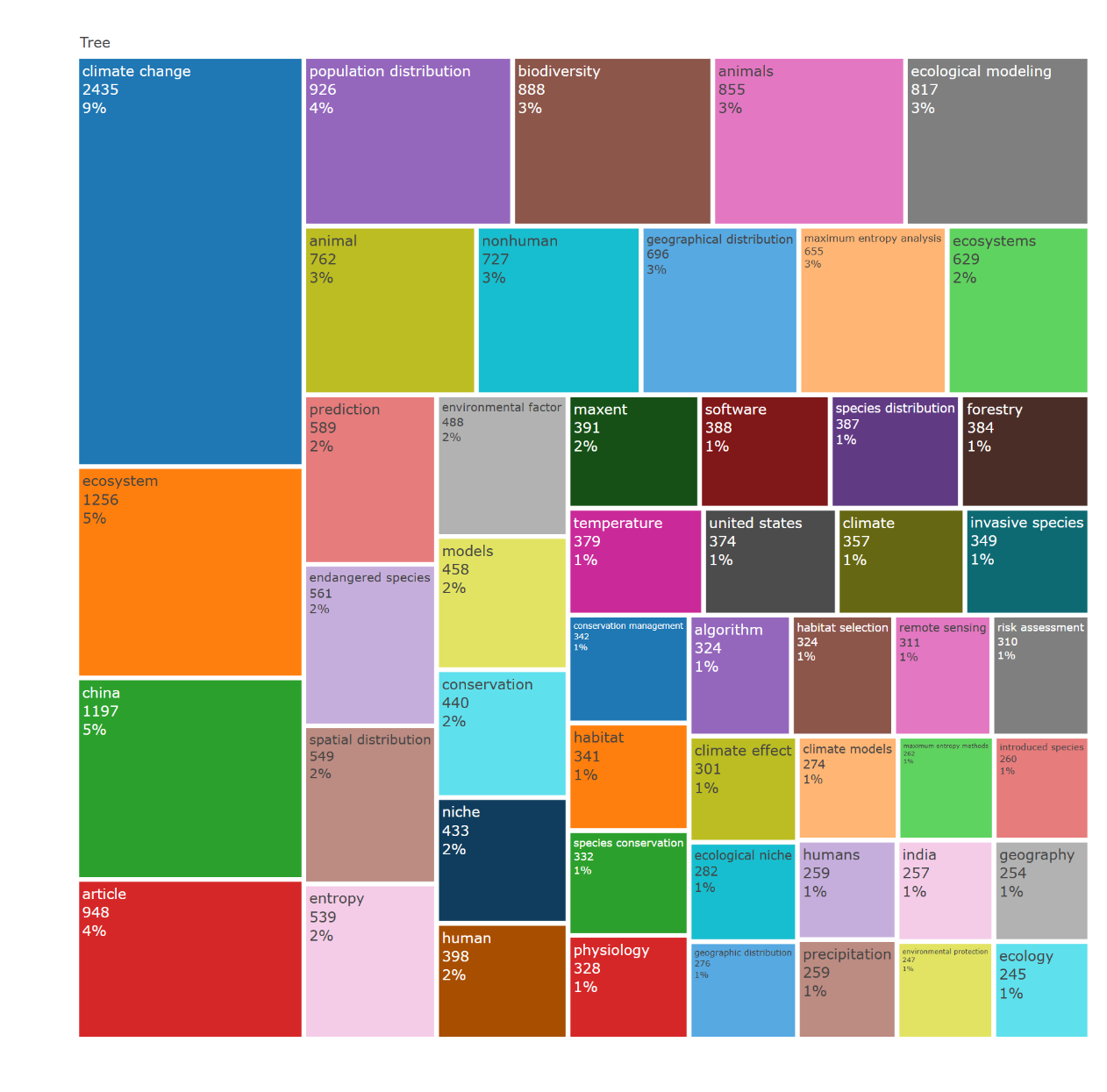


**Figure** **S3.** *Keyword Plus Analysis for Maxent Publications; this treemap visualizes the most frequent "keyword plus" terms associated with Maxent-related scientific publications. The size of each rectangle is proportional to the frequency of the keyword, indicating key research themes and areas of application for the Maxent model.*


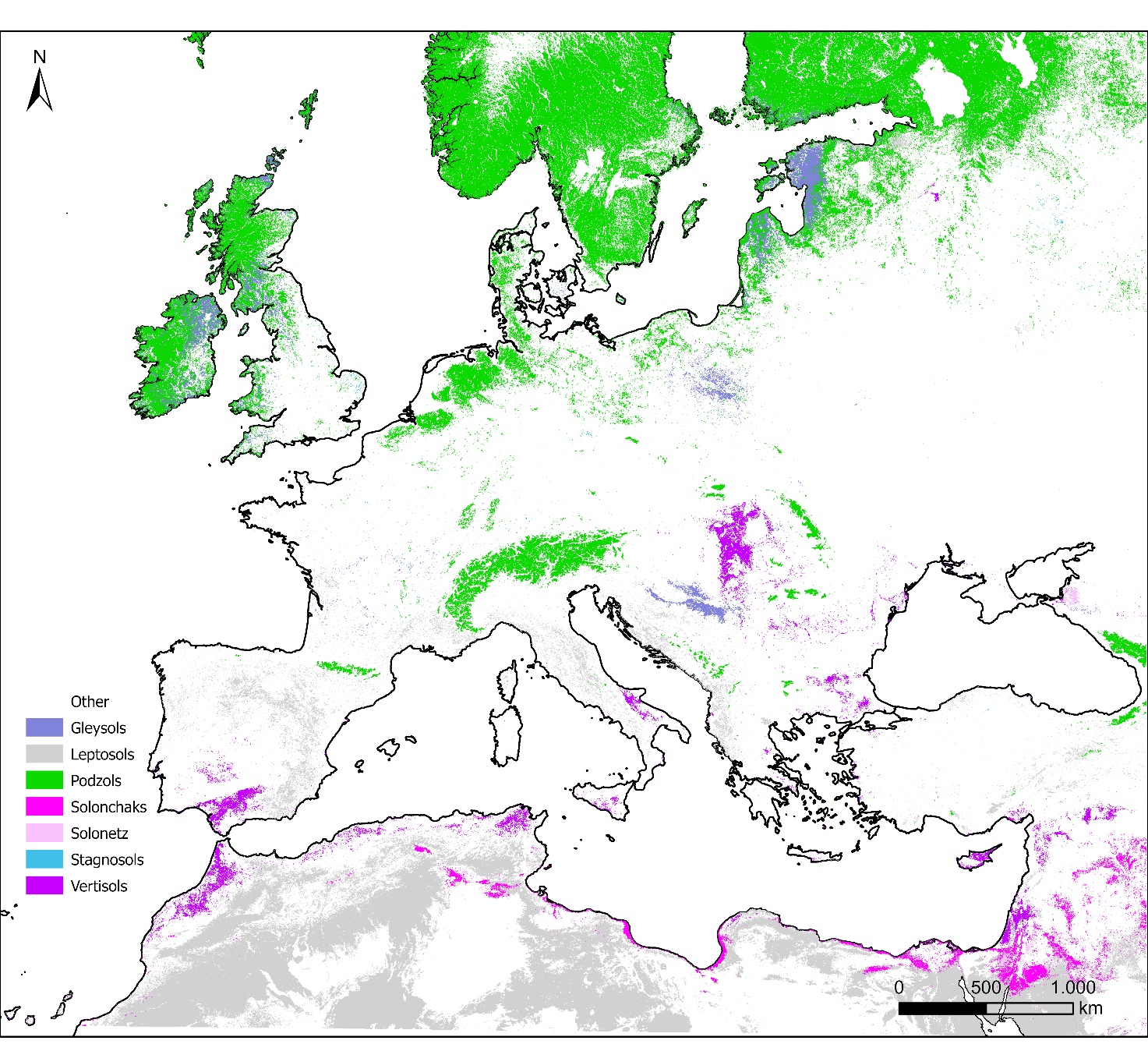


**Figure S4.** *Potential Unsuitable Soil Groups for Olive Cultivation in the Mediterranean Basin This map identifies areas with soil types considered unsuitable for olive cultivation based on the World Reference Base (WRB) 2022 classification system. Different colors represent various unsuitable soil groups (e.g., Gleysols, Leptosols, Podzols, Solonchaks, Solonetz, Stagnosols, Vertisols)*


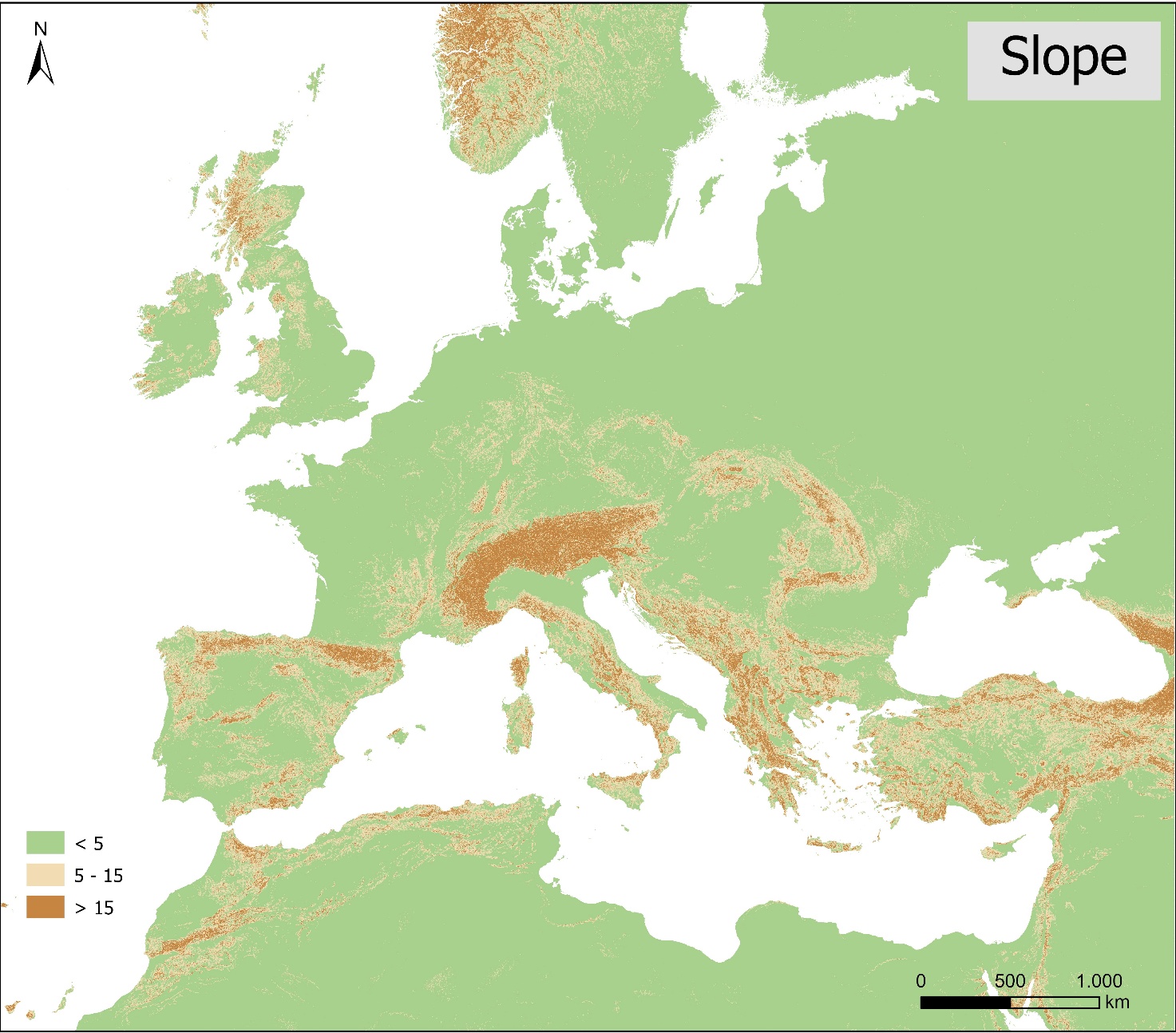


**Figure S5.** *Slope Map for the Mediterranean Basin, expressed as a percentage. Areas are categorized into three slope classes: < 5% (green), 5-15% (light brown), and > 15% (dark brown), providing insights into topographical limitations for olive cultivation*


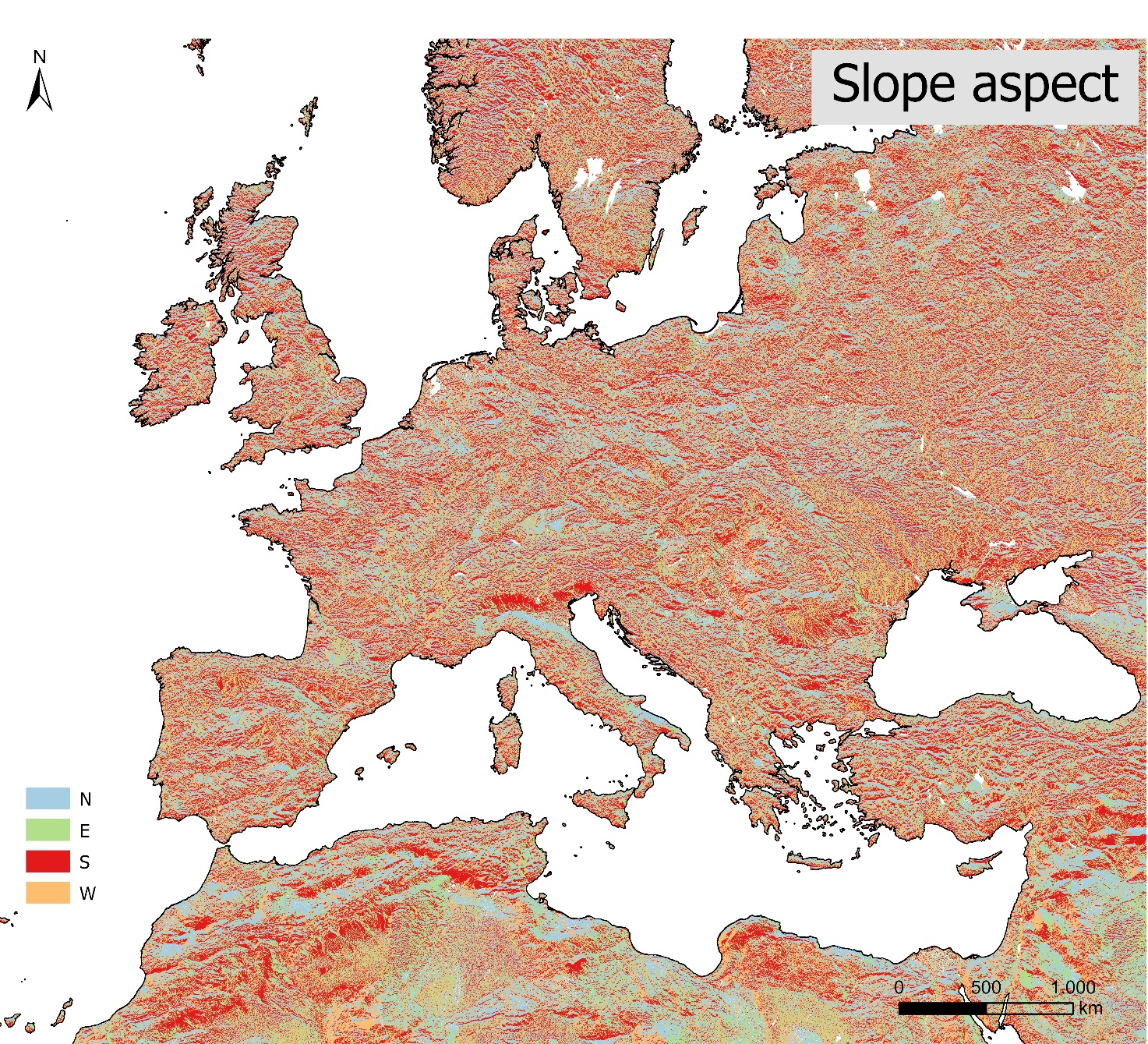


**Figure S6.** *Slope Aspect Map for the Mediterranean Basin*


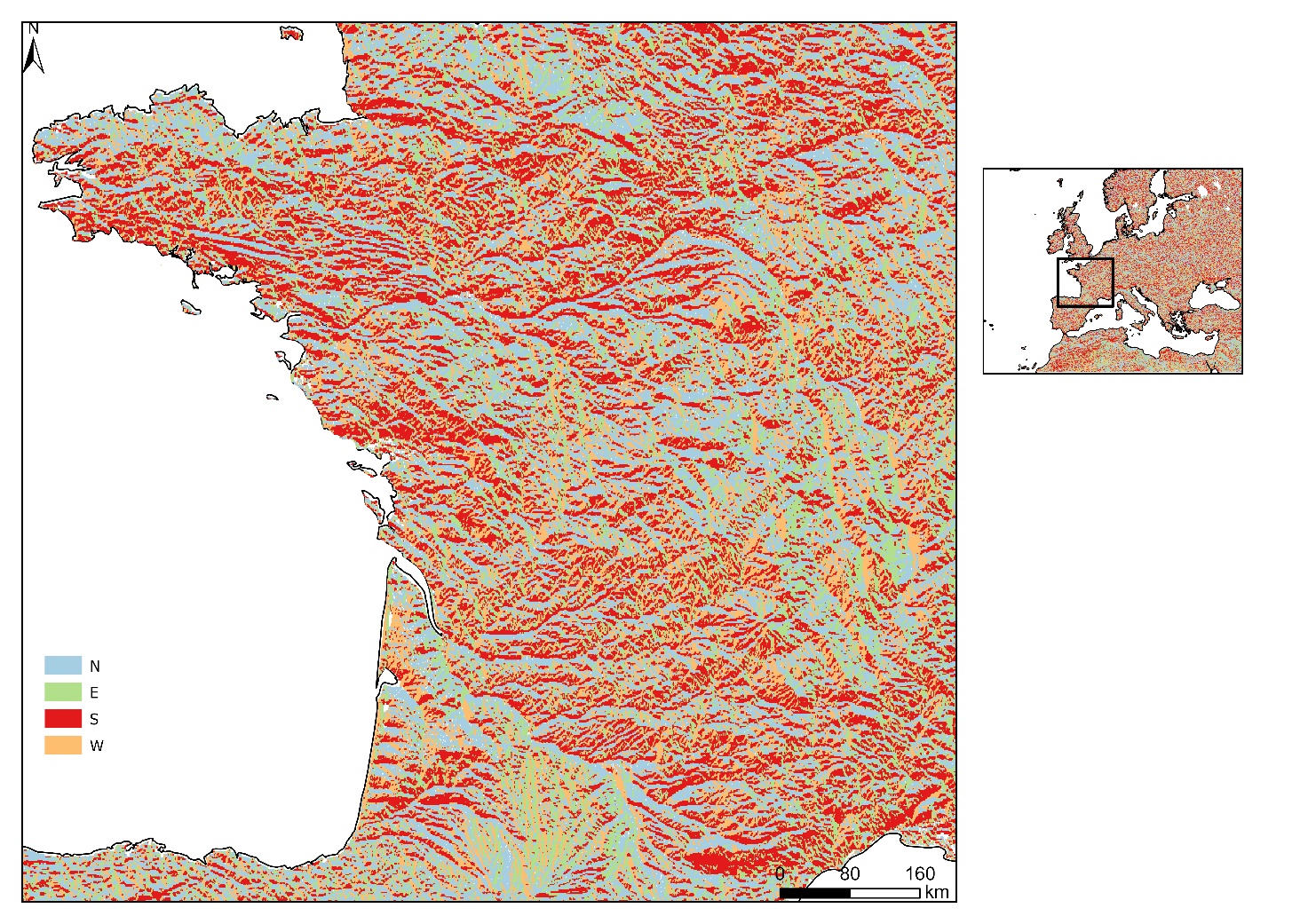


**Figure S7.** *Detailed map focuses on the slope aspect within France, highlighting the directional orientation of slopes (North, East, South, West). This region is considered particularly promising for future olive cultivation, and understanding its slope aspect is crucial for optimizing new olive groves*

**References**

Aria, M., Cuccurullo, C., 2017. bibliometrix: An R-tool for comprehensive science mapping analysis. J. Informetr. 11(4), 959–975. <https://doi.org/10.1016/j.joi.2017.08.007>

*
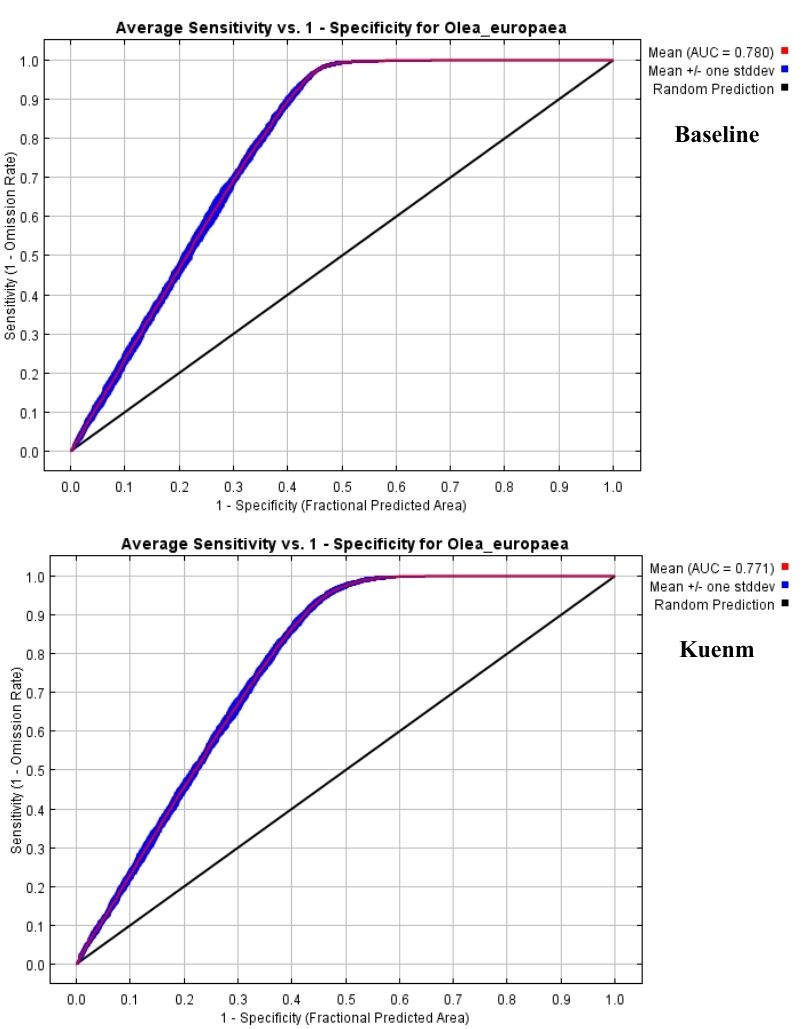
***Figure S8.** *Average Receiver Operating Characteristic (ROC) curve from the replicated MaxEnt runs. Upper panel = baseline MaxEnt; lower panel = kuenm model*

**Figure S9.** *Jackknife test of variable importance for the MaxEnt model. Model gain reflects how much better the model predicts species presence compared to a uniform distribution; higher gain indicates stronger explanatory power. Bars show gain when each variable is used in isolation (blue), excluded (turquoise), or included with all others (red). Left panel = Baseline MaxEnt; Right panel = Kuenm*

**
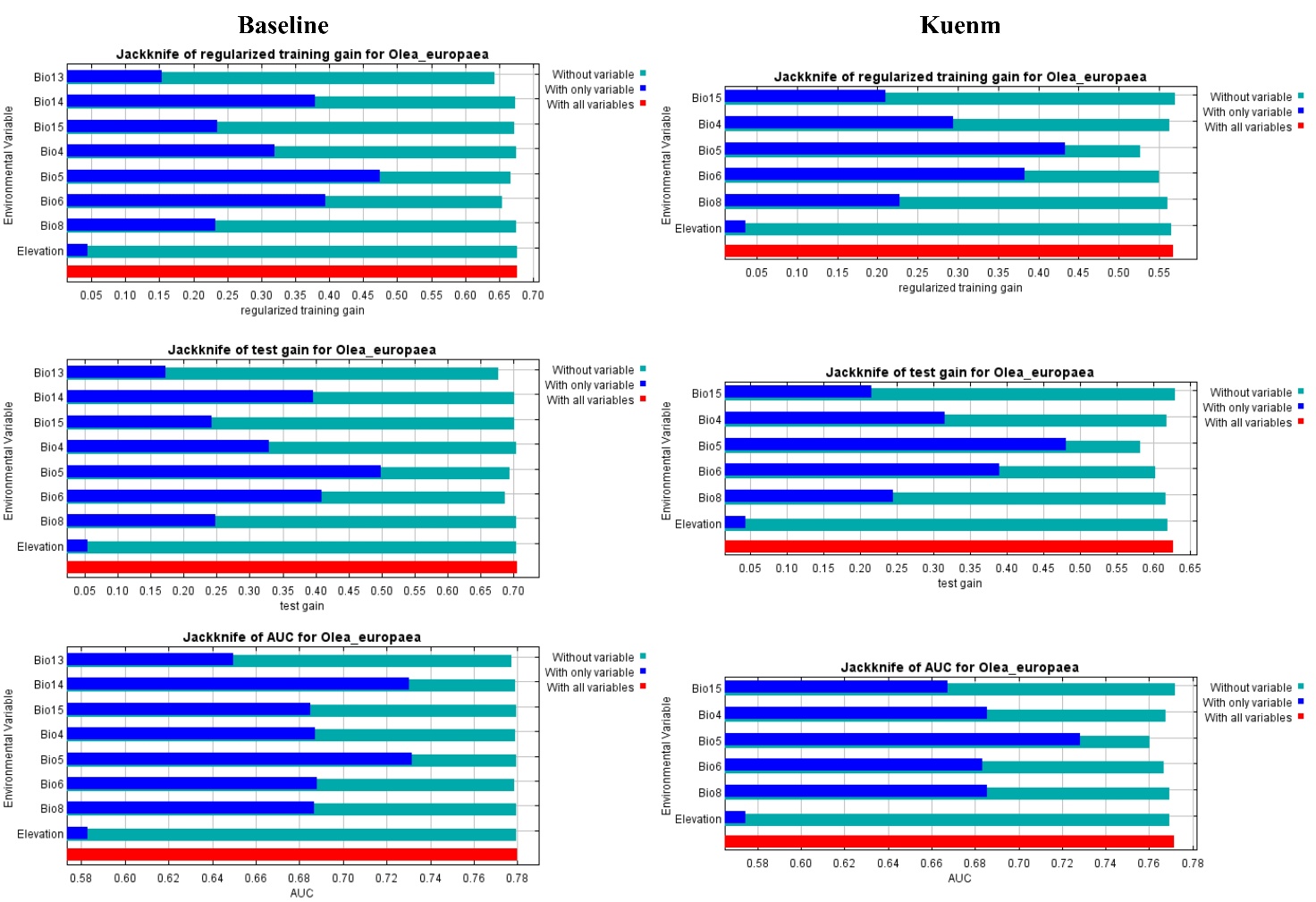
**

**Table S1**. Descriptive statistics of the Aridity Index (AI) across suitable and unsuitable areas for each climate scenario. Includes means, quartiles, and standard deviation.

| scenario | suitability | t test | mean | median | sd | min | max | q25 | q75 | n |
| --- | --- | --- | --- | --- | --- | --- | --- | --- | --- | --- |
| Actual | 0 | 858.39 | 0.67 | 0.71 | 0.54 | 0.00 | 3.00 | 0.07 | 1.02 | 16167131 |
| Actual | 1 |  | 0.49 | 0.44 | 0.26 | 0.07 | 2.63 | 0.30 | 0.62 | 2466977 |
| SSP126 | 0 | 693.41 | 0.63 | 0.65 | 0.52 | 0.00 | 3.00 | 0.06 | 0.97 | 15682223 |
| SSP126 | 1 |  | 0.48 | 0.42 | 0.28 | 0.03 | 2.46 | 0.28 | 0.63 | 2978797 |
| SSP585 | 0 | 450.96 | 0.60 | 0.61 | 0.51 | 0.00 | 3.00 | 0.05 | 0.93 | 15355358 |
| SSP585 | 1 |  | 0.50 | 0.44 | 0.29 | 0.03 | 2.66 | 0.29 | 0.68 | 3314023 |

**
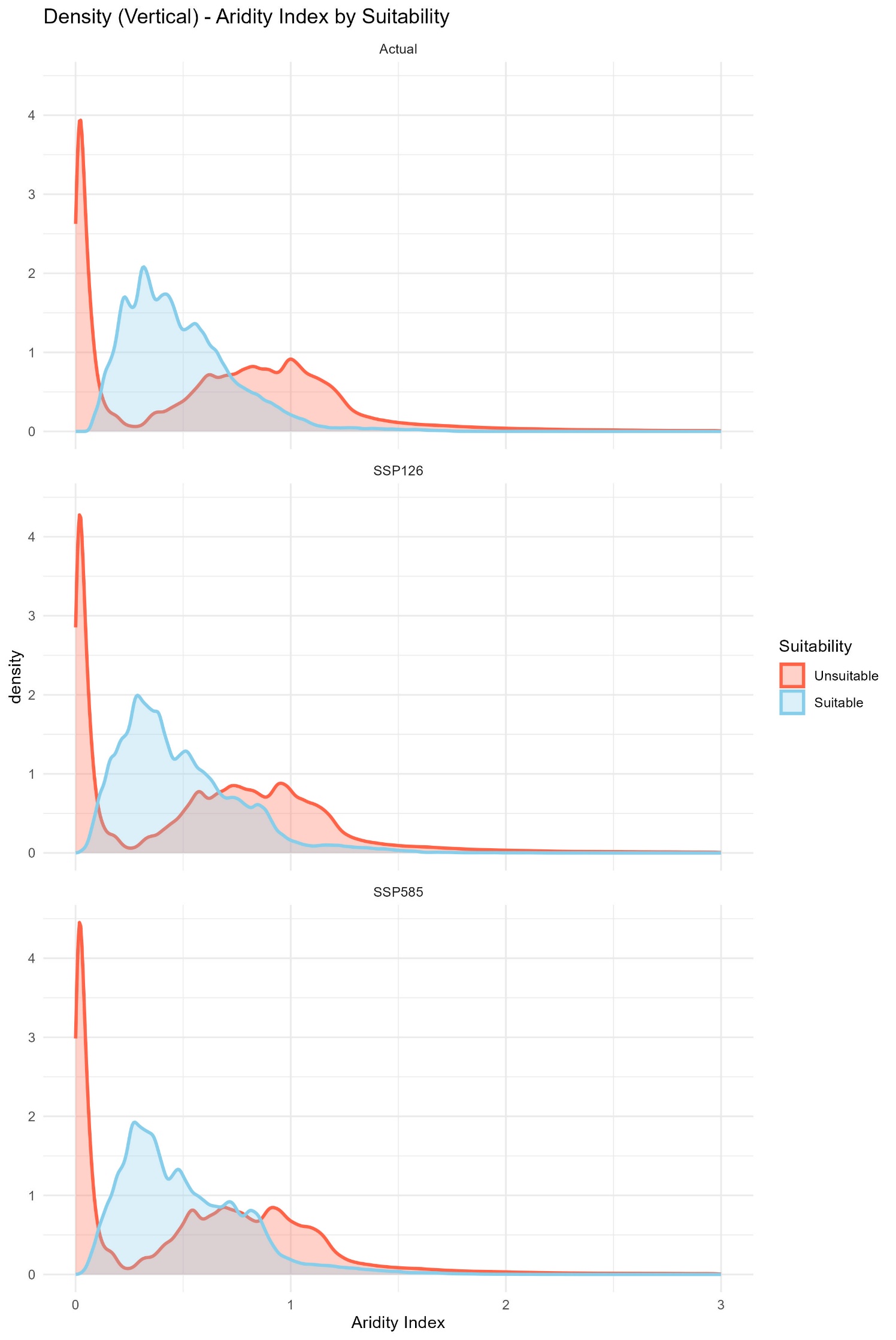
**

**Figure S10**. Density plot for Aridity Index (AI) values by olive suitability for 1) actual; 2) first future scenario ssp126; 3) first future scenario ssp585
